# The Porto European Cancer Research Summit 2021

**DOI:** 10.1002/1878-0261.13078

**Published:** 2021-09-13

**Authors:** Ulrik Ringborg, Anton Berns, Julio E. Celis, Manuel Heitor, Josep Tabernero, Joachim Schüz, Michael Baumann, Rui Henrique, Matti Aapro, Partha Basu, Regina Beets‐Tan, Benjamin Besse, Fátima Cardoso, Fátima Carneiro, Guy van den Eede, Alexander Eggermont, Stefan Fröhling, Susan Galbraith, Elena Garralda, Douglas Hanahan, Thomas Hofmarcher, Bengt Jönsson, Olli Kallioniemi, Miklós Kásler, Eva Kondorosi, Jan Korbel, Denis Lacombe, José Carlos Machado, José M. Martin‐Moreno, Francoise Meunier, Péter Nagy, Paolo Nuciforo, Simon Oberst, Júlio Oliveiera, Maria Papatriantafyllou, Walter Ricciardi, Alexander Roediger, Bettina Ryll, Richard Schilsky, Grazia Scocca, Raquel Seruca, Marta Soares, Karen Steindorf, Vincenzo Valentini, Emile Voest, Elisabete Weiderpass, Nils Wilking, Amanda Wren, Laurence Zitvogel

**Affiliations:** ^1^ Cancer Center Karolinska Karolinska University Hospital Stockholm Sweden; ^2^ European Academy of Cancer Sciences Stockholm Sweden; ^3^ The Netherlands Cancer Institute Amsterdam The Netherlands; ^4^ Danish Cancer Society Research Centre Copenhagen Denmark; ^5^ Ministry for Science, Technology and Higher Education Lisbon Portugal; ^6^ Vall d’Hebron University Hospital Vall d´Hebron Institute of Oncology (VHIO) Barcelona Spain; ^7^ Cancer Core Europe Amsterdam The Netherlands; ^8^ International Agency for Research on Cancer (IARC/WHO) Lyon France; ^9^ Cancer Prevention Europe Lyon France; ^10^ German Cancer Research Center (DKFZ), and National Center for Tumor Diseases (NCT) Heidelberg Heidelberg Germany; ^11^ Portuguese Oncology Institute of Porto Porto Comprehensive Cancer Centre (P.CCC) Porto Portugal; ^12^ The European Cancer Organisation (ECO) Brussels Belgium; ^13^ Gustave Roussy Cancer Campus Grand Paris Villejuif France; ^14^ Champalimaud Clinical Center/Champalimaud Foundation Lisbon Portugal; ^15^ Faculty of Medicine University of Porto/Centro Hospitalar Universitário de São João & Ipatimup/i3S Porto Portugal; ^16^ European Commission Joint Research Centre (JRC) Geel Belgium; ^17^ Princess Máxima Center for Paediatric Oncology & University Medical Center Utrecht The Netherlands; ^18^ AstraZeneca Cambridge UK; ^19^ Swiss Cancer Center Leman (SCCL) Lausanne Switzerland; ^20^ Lund University and IHE Lund Sweden; ^21^ Stockholm School of Economics Stockholm Sweden; ^22^ Science for Life Laboratory Stockholm Sweden; ^23^ Ministry of Human Resources Budapest Hungary; ^24^ European Commission’s Group of Chief Scientific Advisors Brussels Belgium; ^25^ European Molecular Biology Laboratory Heidelberg Germany; ^26^ EORTC Headquarters Brussels Belgium; ^27^ Institute for Investigation and Innovation in Health (i3S)/Porto Comprehensive Cancer Centre (P.CCC) Porto Portugal; ^28^ University of Valencia Valencia Spain; ^29^ Fédération of European Academies of Medicine Brussels Belgium; ^30^ National Institute of Oncology Budapest Hungary; ^31^ Cancer Research UK Cambridge Centre Cambridge UK; ^32^ Organisation of European Cancer Institutes (OECI) Brussels Belgium; ^33^ Molecular Oncology Editorial Office Heidelberg Germany; ^34^ Università Cattolica del Sacro Cuore Rome Italy; ^35^ European Federation of Pharmaceutical Industries and Associations Brussels Belgium; ^36^ MSD International Business GmbH Kriens Switzerland; ^37^ Melanoma Patient Network Europe Sweden; ^38^ Past President and former Chief Medical Officer of the American Society of Clinical Oncology Alexandria VA USA; ^39^ European Cancer Patient Coalition Brussels Belgium; ^40^ Policlinico Gemelli Rome Italy; ^41^ Karolinska Institutet Stockholm Sweden

**Keywords:** Cancer Mission, cancer research/care/prevention continuum, clinical/prevention trials, comprehensive cancer centres, infrastructures for translational cancer research, outcomes research, science policy

## Abstract

Key stakeholders from the cancer research continuum met in May 2021 at the European Cancer Research Summit in Porto to discuss priorities and specific action points required for the successful implementation of the European Cancer Mission and Europe's Beating Cancer Plan (EBCP). Speakers presented a unified view about the need to establish high‐quality, networked infrastructures to decrease cancer incidence, increase the cure rate, improve patient's survival and quality of life, and deal with research and care inequalities across the European Union (EU). These infrastructures, featuring Comprehensive Cancer Centres (CCCs) as key components, will integrate care, prevention and research across the entire cancer continuum to support the development of personalized/precision cancer medicine in Europe. The three pillars of the recommended European infrastructures – namely translational research, clinical/prevention trials and outcomes research – were pondered at length. Speakers addressing the future needs of translational research focused on the prospects of multiomics assisted preclinical research, progress in Molecular and Digital Pathology, immunotherapy, liquid biopsy and science data. The clinical/prevention trial session presented the requirements for next‐generation, multicentric trials entailing unified strategies for patient stratification, imaging, and biospecimen acquisition and storage. The third session highlighted the need for establishing outcomes research infrastructures to cover primary prevention, early detection, clinical effectiveness of innovations, health‐related quality‐of‐life assessment, survivorship research and health economics. An important outcome of the Summit was the presentation of the Porto Declaration, which called for a collective and committed action throughout Europe to develop the cancer research infrastructures indispensable for fostering innovation and decreasing inequalities within and between member states. Moreover, the Summit guidelines will assist decision making in the context of a unique EU‐wide cancer initiative that, if expertly implemented, will decrease the cancer death toll and improve the quality of life of those confronted with cancer, and this is carried out at an affordable cost.

## Graphical abstract

Key stakeholders from the cancer research continuum met at the European Cancer Research Summit to discuss priorities for the successful implementation of Cancer Mission and Europe's Beating Cancer Plan. The resulting Porto Declaration called for a collective action to develop cancer research infrastructures, foster innovation and decrease inequalities. Here, we present the Summit recommendations for an EU‐wide cancer initiative that, if expertly implemented, will decrease the cancer death toll and improve the quality of life of cancer patients and survivors.

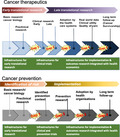

**Abbreviations**
AI: artificial intelligence
BECA: Special Committee on Beating Cancer of the European Parliament
BoB: Basket of Baskets (trial)
CCC: Comprehensive Cancer Centre
CCE: Cancer Core Europe
CEEAO: Central–Eastern European Academy of Oncology
DART: Data Rich Clinical Trials
DKFZ: German Cancer Research Center
DKTK: German Cancer Consortium
DRUP: Drug Rediscovery Protocol
EACS: European Academy of Cancer Sciences
EBCP: Europe´s Beating Cancer Plan
ECAC: European Code Against Cancer
ECPC: European Cancer Patient Coalition
EFPIA: European Federation of Pharmaceutical Industries and Associations
EMBL: European Molecular Biology Laboratory
EORTC: European Organisation for Research and Treatment of Cancer
ERN: European Reference Network
ESMO: European Society of Medical Oncology
IARC: International Agency for Research on Cancer
IMI: Innovative Medicine Initiative
IQN: Path International Quality Network for Pathology
JRC: Joint Research Centre EU
MRI: magnetic resonance imaging
MTB: Molecular Tumour Board
NCT: National Center for Tumor Diseases
NGS: next‐generation sequencing
OECI: Organisation of European Cancer Institutes
QoL: health‐related quality of life
RCT: randomized clinical trial
RT: radiation therapy
VBHC: value‐based health care
WGS: whole‐genome sequencing
WSI: whole‐slide images

## 1 Introduction

The effective implementation of the EU cancer research strategy has been the focus of the European Cancer Research Summit, which took place in Porto in May 2021 and mainly discussed the requirements for distributed and interconnected infrastructures needed to support research on cancer therapeutics, care and prevention [1]. The European Cancer Summit and the resulting Porto Declaration on cancer research [2] stemmed from the previous ‘Europe: Unite against Cancer’ Declaration that was signed by the consecutive German, Portuguese and Slovenian EU presidencies in October 2020, with the aim to outline future directions for cancer research and care throughout Europe [3]. This initiative prepared the grounds for European organizations and stakeholders to determine a common strategy for effectively delivering equal care to European cancer patients.

As an integral part of the Horizon Europe Framework Programme for Research and Innovation (2021‐2027), a set of European Research and Innovation Missions aim to deliver solutions to some of the greatest challenges facing Europe, including cancer. As highlighted by one of the Summit speakers, Guy van den Eede, cancer accounts for more deaths than any other disease in the age group of below 65 in the EU. While this geographical area is home to < 10% of the world’s population, it collects 23% of all cancer cases. On average, only one in two cancer patients survive, and one in two of us will face cancer in our lifetimes. In economic terms, cancer costs the EU almost €97 billion in 2018. In futuristic terms, several factors, including the EU’s ageing population, will see that all numbers and costs increase unless serious action is taken, such as doing more on prevention, early detection, quality of care and more [4].

In the context of Horizon Europe, both the Cancer Mission [5, 6, 7] and Europe’s Beating Cancer Plan (EBCP) [8] have highlighted the impactful commitment of policymakers to unite European countries in their efforts to substantially reduce the enormous cancer burden. Common aim is to decrease mortality and improve patients' health‐related quality of life by promoting cost‐effective, evidence‐based best practices in cancer prevention, treatment and care. Addressing these challenges will require concerted actions across the whole cancer research/care/prevention continuum that spans from basic and preclinical research to clinical and prevention research and outcomes research [9].

In this landscape, policymakers, academic researchers, patient representatives and pharmaceutical industry members contributed to keynote sessions and panel discussions at the European Cancer Research Summit 2021. Rui Henrique, the main organizer, and Julio E. Celis, the Chair of the Scientific Advisory Committee, welcomed the Summit participants. The Summit started with a brief glimpse over current developments in the European cancer policy landscape. It continued with distilling the view of the cancer research community on the basic requirements that will enable the implementation of an effective European Cancer Mission. Finally, it focused on the specific recommendations by key stakeholders to establish efficient infrastructures for translational research, clinical/prevention trials and outcomes research. Panel discussions complemented the perspectives of key lectures and set the scene for a multivoiced, yet highly collaborative, pan‐European initiative to tackle the challenges of cancer for the individual, the health systems and the society.

## 2 A broad glimpse into the current European cancer policy landscape

The Summit´s first session focused on current and future European cancer policy plans, in which collaboration among policymakers, scientists and patient organizations is deemed indispensable. The EU Commissioner for Health and Food Safety, **Stella Kyriakides**, opened the session by thanking the cancer community for supporting EBCP, which in partnership with the European Cancer Mission will address current challenges in cancer research, prevention and care. Ms Kyriakides highlighted the importance of interdisciplinary collaboration and emphasized the need for evidence‐based knowledge and its translation into policy and political decisions. The planned EU initiatives for fostering cancer research, prevention and care will financially be supported partly by EU4Health, but, as Ms Kyriakides noted, the clear commitment of all EU member states would be equally crucial. In addition, European Comprehensive Cancer Centres (CCCs) and the European Parliament Special Committee on Beating Cancer (BECA) will have key parts in these efforts – the concrete contribution of BECA being currently under discussion.

Further corroborating the message of Stella Kyriakides on the importance of collaboration, **Marta Temido**, Minister for Health, Portugal, recognized that public health authorities alone could not tackle the major societal challenge of cancer. Ms Temido highlighted the need to invest in research and technology using a research‐driven, patient‐centred approach and mentioned that BECA would support efforts across the EU. While clinical research and the national cancer plan are key priorities of the Portuguese government, they should also be prioritized by all other EU member states, indicated Ms Temido, who also encouraged EU member states to sign the ‘Porto Declaration’.

**Mariya Gabriel**, EU Commissioner for Innovation, Research, Culture, Education & Youth, next informed the audience that the health cluster of Horizon Europe features a Cancer Mission with well‐defined goals as a key priority, aiming at improving prevention, diagnosis and treatment. While the COVID‐19 pandemic caused delays in cancer research and weakened European Research Networks (ERNs), Horizon Europe is expected to restrengthen partnerships in health, such as the partnership on personalized medicine, innovative health initiatives that build on the success of the Innovative Medicine Initiative (IMI), and the European Institute of Innovation and Technology Health. According to the Commissioner, work on the Cancer Mission implementation plan is currently underway; the next is to invigorate EU member states and regional funders. Marie Sklodowska‐Curie actions will also help build scientific excellence and cooperation across countries. The new EC Knowledge Centre on Cancer, launched on 30 June 2021, is expected to coordinate the efforts of EU member states [4]. In addition, prevention is a key priority of the EU, and further actions are being considered to promote a healthy lifestyle across Europe, including the Healthy Life Style for all programme (yet to be launched).

Focusing on one of the points briefly introduced by Stella Kyriakides, namely the role of European CCCs in a Cancer Mission, **Manuel Heitor**, Minister for Science, Technology and Higher Education, Portugal, emphasized the need to engage European‐wide network of CCCs and infrastructures effectively. Mr Heitor highlighted the need to ensure strong and widely accessible networks composed of infrastructures for three research directions: translational research, clinical/prevention trials and outcomes research. In addition, Manuel Heitor argued that the effective implementation of a European Cancer Mission would help reduce the current gap between science and policy. This is necessary to achieve the target of ensuring a long‐life expectancy for three out of four newly diagnosed cancer patients by 2030 across Europe. The latter will require the active involvement of all European communities involved in cancer research and cancer prevention/health care, as well as of cancer patient organizations in policymaking, to align specific scientific and diversified local issues into an overall strategy with practical relevance to all European citizens at large [10].

## 3 Route to the Cancer Mission: a shared view of the cancer community

### 3.1 The perspective of the European Academy of Cancer Sciences

**Anton Berns**, President of the European Academy of Cancer Sciences (EACS), discussed the instrumental role that the EACS played in placing cancer research on the European Agenda and identified the issues that needed attention. In collaboration with a large number of European cancer organizations, the views have been voiced [7]; evidently, we face a major societal challenge with a substantial rise in incidence and projected deaths from cancer in the coming decades. Not only will more patients develop cancer, but many more will also be living with cancer, which makes cancer one of the main chronic diseases. It will also lead to increasing demand for personnel and skyrocketing costs. Unfortunately, we will lack both the workforce and the funds for this. At the same time, we have to rectify the inequalities in access to cancer care between and within the EU countries.

EACS believes that the Cancer Mission can provide an important stimulus to tackle this problem if reachable goals are defined and funds are spent wisely. Thus, the ambition that 75% of patients diagnosed with cancer in 2030 survive 10 years or longer with a good quality of life may come within reach.

**Fig. 1:**
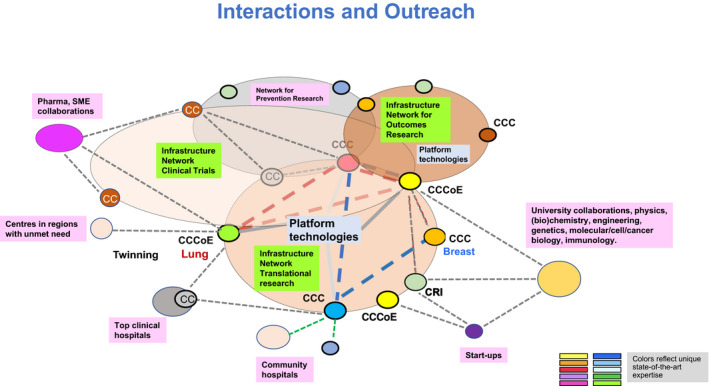
Research networks to reach the critical mass for innovative translational cancer research aiming at personalized/precision cancer medicine: the necessary infrastructures, patients, biological samples, specific technological resources and expertise.

To achieve this, Anton Berns outlined the need to strengthen the complete continuum of cancer research (Fig. 1), from better understanding the underlying biology to implementing new interventions and making cancer care and prevention cost‐effective (Box 1).

Box 1 **Steps towards strengthening the complete continuum of cancer research**

Efforts to implement an effective European Cancer Mission that will achieve well‐defined goals in terms of cancer care and cancer prevention require research in several areas:

- Identification of individuals at risk (carcinogen exposure, lifestyle, socio‐economics, genetic predisposition).
- New prevention strategies (medical prevention, vaccination, encouraging healthy lifestyles).
- New early detection strategies based on better understanding the biology of malignant disease (leading to cost‐effective screenings methods with proven benefit for patients).
- Precision medicines (more cures, treatments tailored to individual patients).
- Patient in central position (quality of life, physical, psychosocial and socio‐economic aspects).
- Outcomes research (assess benefits for patients and cost‐effectiveness).
- Implementation research to facilitate both the swift introduction of and equal access to proven effective interventions.

These activities are best embedded in infrastructures with sufficient critical mass focusing on basic/ translational research, clinical research and outcomes research.

There is also the need for a number of more specific measures:

- Stimulate paediatric and geriatric oncology.
- Install an expert board to advise on legal issues (carcinogen exposure reduction, data sharing, and socio‐economics).
- Incentivize centres to acquire critical mass and to commit to quality standards: support accreditation programmes for Comprehensive Cancer Centres (CCCs) by the Organization of European Cancer Institutes (OECI) and the German Cancer Aid and Designation of CCCs of Excellence by assessment of translational research by EACS and enable the sustainability of networks between such centres (Fig. 1).
- Provide tailored support to centres in areas with unmet need and facilitate their ‘twinning’ with expert centres.
- Encourage outreach of CCCs and networks to other stakeholders (hospitals, patient organizations and industry).
- Support CCCs and professional societies to educate and train the next generation of cancer researchers and cancer specialists (capacity building).

The Cancer Mission is in the view of the EACS best served by bottom‐up incentives tuned to the strengthening of infrastructures and stimulating innovative research in all the domains of the research continuum, whereby quality is a requirement to receive funding. Furthermore, since real innovation and breakthroughs primarily result from original ideas of creative investigators, it will be critical to support innovative principal investigator (PI)‐led research projects and PI‐initiated early phase clinical trials. The EACS firmly believes that next to supporting the establishment of infrastructures and networks, ERC (European Research Council) or Synergy ERC‐like Funding to stimulate the specific areas in the cancer research continuum could serve as an important cornerstone of the Cancer Mission.

### 3.2 Report from the Cancer Mission Board

While designing the Mission on Cancer, the European Commission (EC) invited a Board of European experts – covering cancer research, innovation, policy, healthcare provision and practice – to define an ambitious and measurable goal with a substantial impact on and relevance for society and citizens of Europe. The EC also asked the Board to propose a coherent set of actions to achieve this goal in a set time frame. These actions will be implemented through Horizon Europe and other EU and its member states instruments and aligned with other initiatives at the EU and member state level.

**Walter Ricciardi**, President of the EU Cancer Mission Board, presented the EU Cancer Mission Board report, which explains how a mission‐driven approach can save and improve the lives of millions of European citizens exposed to cancer and/or cancer risk factors. This report sets out the Mission`s goal on cancer and makes recommendations on how to achieve this goal.

In finalizing this Mission report, the Board was assisted by the Cancer Mission Assembly and by inputs from a wide network of experts and organizations (academic, private sectors and advocacy groups). In addition, the Board received feedback from the 27 member states, members of the European Parliament and several Directorates‐General of the EC, as well as from a number of consultation and engagement sessions with EU citizens, cancer patients and survivors organized in their countries and native language or online meetings with participants from across the entire EU.

In accordance with what was highlighted by the EU Commissioners above, the Cancer Mission Board report [6] will be used as a basis for further stakeholder and citizen engagement activities and define a broad strategy for the first four years of the Horizon Europe Programme. In addition, synergies will be developed with national cancer plans and other actions of member states, with the activities of other Horizon Europe Missions and research and investment programmes, as well as with other EU policies and actions, particularly the EBCP.

The report indicates that given the high level of ambition, a comprehensive plan of bold actions supported by all member states and stakeholders – including patients, survivors, carers and the wider public – is required to achieve the Mission’s goal. Effective interventions are needed to develop the three pillars of the EU Cancer Mission: (a) prevention; (b) diagnostics and treatment of cancer; and (c) the quality of life of cancer patients, survivors, and their families and carers. Effective interventions in these areas require a thorough understanding of cancers, causal factors and mechanisms, and their impact, and this understanding emerges as the basis for actions. Furthermore, effective policy measures are needed. Resources should be allocated to ensure that citizens and other stakeholders in all EU member states have equitable access to high‐quality prevention, diagnostics and treatment, care and support, including access to research funding and knowledge. Finally, as underscored in the Mazzucato report ‘Governing missions in the European Union’ [11] the mission‐oriented process’s success will lie in the set‐up of novel flexible governing structures to correctly balance with effective portfolio management that enables cross‐sectoral and cross‐institutional coordination.

### 3.3 The cancer research continuum

**Fig. 2:**
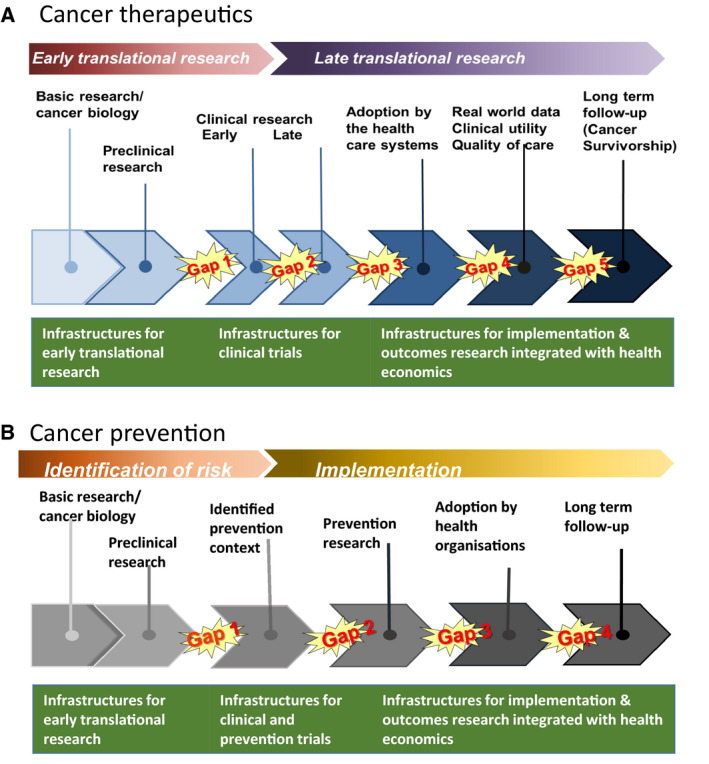
Cancer research continuum. Gaps in the therapeutics (A) and prevention (B) cancer research need to be integrated, hopefully through the establishment of infrastructures for early translational research, clinical and prevention trials and outcomes research (adapted from ref. 9).

Reflecting Anton Berns' presentation and the three pillars of the Mission highlighted in the Cancer Mission Board report, **Ulrik Ringborg**, Secretary‐General of the EACS, next emphasized that a Mission on Cancer must cover the entire cancer research continuum [9]. For therapeutic research, the continuum starts with basic/preclinical research and proceeds to clinical and outcomes research, including long‐term follow‐up (Fig. 2A). Translational research aims at bridging a number of gaps in the continuum, the most important between basic/preclinical and early clinical trials and between outcomes of clinical trials and implementation into health care. The research continuum is representative of all components of therapeutics and cancer care. For example, precision cancer medicine is often discussed for medical treatment with targeted drugs but has the same relevance for all treatment modalities such as surgery, radiation therapy, chemotherapy or immunotherapy. The research continuum is similar for prevention with a number of gaps, among them the important implementation research (Fig. 2B). Translational research is bidirectional since research questions are identified in the clinical/prevention part of the research continuum but have to be answered by basic/preclinical research.

Next, the Summit focused on infrastructures for translational cancer research covering the research continuum, in other words, how to conduct the research required to reach the goals. As shown in Fig. 2, three main infrastructures are needed, and these were discussed in three sessions, each focusing on: (a) translational research with main focus on early translational research; (b) clinical/prevention trials; and (c) outcomes research.

## 4 Infrastructure for translational research

The session on key infrastructure components of early translational cancer research focused on: basic/preclinical research; Molecular and Digital Pathology; Immunotherapy; Liquid Biopsies; and Data Science. Speakers discussed how the above components can bring innovation in clinical and prevention research. CCC‐based networks, required to reach the critical mass for personalized/precision cancer medicine, were also approached.

### 4.1 Basic/preclinical research: generating proof‐of‐concept clinical trials is the engine for translational research

**Alexander Eggermont**, Princess Máxima Center for Pediatric Oncology and University Medical Center, Utrecht, the Netherlands, highlighted the unparalleled analytic power of current translational research as compared to the research analytic power observed 5–10 years ago, as a result of a myriad of new high‐technology platforms that allow for rapid characterization of relevant tumour components. Current technologies can, for example, shed light onto the components of single tumour cells, tumour heterogeneity and clonal evolution, tumour metabolism, the tumour microenvironment (TME), the TME structural and immune components, and TME evolution over time, or in response to treatments. In detail, single‐cell sequencing can be used to unravel the various malignant or immune components of a tumour, as well as modifications brought over by immunomodulatory, chemotherapeutic or targeted drugs. In parallel, multiomics approaches enable the rapid evaluation and molecular understanding of drug sensitivity or resistance in new organoid technologies that are superior to other methods or much less time‐consuming and thereby cost‐effective. These advances in combination with the latest preclinical cancer models (Box 2) have accelerated the evaluation of basic research discoveries and their translation in early‐phase clinical trials. Such early clinical trials can generate unparalleled insights into cancer and stimulate the development of cancer therapeutics, provided that translational infrastructures and tailored research programmes are established.

Box 2 **Preclinical cancer models**

**Douglas Hanahan**, Swiss Institute for Experimental Cancer Research (ISREC), Federal Institute of Technology in Lausanne (EPFL), Lausanne, Switzerland, highlighted the need to expedite innovative cancer therapies by leveraging preclinical models. D. Hanahan pointed out that we are in an era of exciting opportunity to markedly improve the detection, diagnosis and treatment of human cancers. An important aspect is the reality that, for most forms of human cancer, there are no ‘magic bullets’ of drugs that produce long‐term remissions/prospective cures and high quality of life. Rather, the possibility for achieving such goals is increasingly appreciated to require multipronged approaches, involving sophisticated combinations that target different identifiable vulnerabilities in the fortresses of tumours to cripple if not destroy cancer. Indeed, knowledge of molecular mechanisms driving tumour growth, underlying crucial vulnerabilities, and adaptive resistance to individual therapies suggests new ways to multitarget tumours, in analogy to conventional warfare, ‘by air, land, and sea’. Importantly, the most effective combinatorial strategies may not necessarily involve simultaneous dosing but rather a precise sequencing of the combinatorial attack, with different drugs being used in other orders and at specific stages of disease progression and responses to the initial therapeutic targeting. The conundrum is that there are many combinations of drugs and sequences to the attack to be accurately and fully tested in human cancer patients. One solution to this challenge is to engage preclinical cancer models, develop hypotheses for novel combinatorial strategies, test the multiplicity of drugs and sequencing of their application, reduce the number of combinations and take the ‘best shots on goal’ into clinical trials. Increasingly sophisticated and accurate models of human cancer have been developed over the past 40 years, largely but not exclusively based on laboratory mice. These include tumour‐derived cancer cells in culture and transplanted into mice to produce tumours, and genetically engineered mice that undergo multistep tumour development and progression to metastatic disease based on signature mutations that define particular human cancers. Other variations include ‘Patient‐Derived Xenograft’ (PDX) tumours and 3‐dimensional ‘organoids’ that more accurately reflect 3‐dimensional tumours and conventional 2D cell cultures. Importantly, with immunotherapy becoming the fourth pillar of cancer therapy, preclinical models need to have active immune systems to evaluate combinations involving drugs that harness the immune system. While current preclinical models are adequate in some cases, there is a clear need to develop improved preclinical models of multiple forms of human cancers.

In the light of the goal of Horizon Europe to change the landscape of cancer therapy, there is a compelling reason to support (a) the use of preclinical cancer models to test novel combinatorial cancer therapeutic strategies aimed to guide patient‐efficient (and more cost‐efficient) clinical trials aimed to assess the efficacy and reveal potentially circumventable – by design – drug resistance mechanisms, and (b) the engineering of next‐generation cancer models that even more accurately recapitulate the dizzying variety of cancer types and subtypes. The visionary integration of preclinical models and therapeutic trials with clinical cancer trials has the potential to enhance the strategic goal of Horizon Europe instrumentally.

A key project in the 2006 sixth framework programme (FP6), the EUROCAN+Plus, provided the first analytic step to overcome the fragmentation of cancer research. In 2008, among other key findings, it was concluded that platforms with critical mass for translational cancer research are needed, and as a result, in 2011 the FP7 EurocanPlatform network of excellence was launched. A direct outcome of this project was the formation of Cancer Core Europe, an alliance between 7 large cancer centres [12] and Cancer Prevention Europe between 10 centres [13].

Similarly, national translational cancer research networks were launched, such as the SIRIC network of eight centres in France. Germany launched the most profound and multilevel comprehensive programme initiatives over the last 12 years. Starting in 2008, a CCC programme launched by the German Cancer Aid led to institute accreditation focusing on multidisciplinary care, translational research infrastructures, clinical trial programme and early‐phase clinical trial infrastructure development (Box 3). This programme created fifteen such CCCs with the overall financial support of over 200 M Euros. Moreover, a federal initiative coordinated by the German Cancer Research Center (DKFZ) was launched in 2012 to finance the German Cancer Consortium (DKTK), a network of 8 CCCs for Translational Cancer Research, with a competitive and well‐funded (35 M per year) programme [14]. In addition, the German Government launched a Multisite National Centres for Tumors (NCT) programme, endowed with over a billion Euros, to expand with four and over time more sites on top of the Heidelberg and Dresden sites to create superstructures with exceptional infrastructures in cancer translational research in collaborative programmes, thus fulfilling elements of the agenda of the federal programme ‘Decade against Cancer’. Overall, these programmes are now leading in Europe and exemplify the dedication it takes to create a structural approach that can make significant and accelerated progress in the fight against cancer.

Box 3 **Reaching critical mass – the German paradigm**

**Stefan Fröhling**, DKFZ/NCT Heidelberg, Heidelberg, Germany, stressed that close collaboration between cancer research centres is required to reach the critical mass necessary for translational research. One important aspect is the sharing of advanced infrastructures for multidimensional characterization of individual cancers regarding their molecular and cellular composition and their functional state and for exploitation of the resulting multilayered data sets. The DKTK has had excellent experience with the collaboration of ten German CCCs organized in eight partner sites. A specific example is the NCT/DKTK MASTER (Molecularly Aided Stratification for Tumor Eradication Research) programme employing genome and RNA sequencing in patients with rare cancers [16]. Here, collaboration within the consortium, particularly the establishment of a common precision oncology workflow, enabled the achievement of relevant patient numbers and the generation of meaningful results, based on which a portfolio of clinical trials is being developed and numerous cross‐site translational research projects are being initiated. DKFZ/NCT Heidelberg is a member of CCE and thus shares infrastructures internationally. CCE provides several infrastructure collaborations, for example the Basket of Baskets study and the Molecular Tumour Board Portal developed at Karolinska Institutet.

In conclusion, translational research infrastructures and programmes are key for the development of tomorrow cancer treatments. This will require adequate investments and collaborative networks through Europe.

### 4.2 The Comprehensive Cancer Centre: an essential infrastructure component

**Simon Oberst**, Cancer Research UK, Cambridge Center, Cambridge, UK, presented on CCCs, organizations where research, care and education cohere and are fully integrated. CCCs do not happen by chance or by virtue of size; they have to be well organized, preferably with a programme structure linking clinicians and researchers around tumour groups or scientific topics. An example of a proven structure is that of the CRUK Cambridge Centre, UK. CCCs pioneer innovation, implement new therapeutic pathways and – if they develop networks around them geographically – can address inequalities (see also detailed comments on this from the panel discussion in Boxes 3, 4, 5). They are thus pivotal to delivering the Cancer Mission and the EBCP.

Simon Oberst also described the complexity of wider Comprehensive Cancer Networks.

Box 4 **Addressing inequalities in cancer research – the Central–Eastern European Academy of Oncology (CEEAO) paradigm**

**Péter Nagy**, The National Institute of Oncology, Budapest, Hungary, and **Miklós Kásler**, Minister of Human Resources, Budapest, Hungary, commented on the role of the Central–Eastern European Academy of Oncology (CEEAO) to address inequalities within the EU and increase the participation of the CEE region in EU‐funded programmes, a pivotal element of the Cancer Mission.

P. Nagy and M. Kásler described their extensive efforts to bring together stakeholders and professionals from oncology care, research and education within the Central‐Eastern European region. A key outcome of these activities was the establishment of CEEAO, so far comprising 22 countries. Leading cancer organizations, such as the EACS, IARC and the Organisation of European Cancer Institutes (OECI), recommend using the CEEAO in the outreach programme of the Cancer Mission. The CEEAO has tight collaborations with the EUhealthSupport, the recommendation 10 subgroup of the cancer mission, the accreditation and designation programme of OECI. Connections with the UNCAN project are being established to lay down the foundations of the Cancer Mission outreach activities. Furthermore, the CEEAO is organizing the first ‘Central–Eastern European Oncology meets Western‐Northern‐Southern European Oncology: Clinical Trial Activities International Conference’, which will bring together leading clinical trial professionals and representatives of major EU organizations in Budapest on the 5–6 November 2021.

The collaborations mentioned above and consortia should be supported to promote the appropriate representation of the Central–Eastern European region in the EBCP initiative.

The twinning programme, which was announced at the 1^st^ Vatican meeting by the president of Cancer Core Europe (CCE), has the potential to further bridge major centres of Western and Central–Eastern member states. The programme builds official, tight, centre‐wide collaborations among CCCs, including sharing infrastructures, designing standardized operation procedures and harmonizing educational activities, clinical trials and translational cancer research programmes. A number of these initiatives have already been launched (e.g. between the DKFZ and the Athens Cancer Centre or the Karolinska Institutet and the Hungarian National Institute of Oncology), so a dedicated programme within the Cancer Mission to support these activities would be highly visible and likely deliver significant outcomes.

As improving the quality of cancer care and research is essential in the Central–Eastern Europe region, novel state‐of‐the‐art infrastructures connecting existing local centres will be needed. To ensure the best utilization of these infrastructures and guarantee high‐level, patient‐centred outcomes, these infrastructures must be established under strict quality control. The consultancy‐based programme by the OECI could help ensure quality.

Recently published data from OECI [15] show that the median research output of CCCs is 4–5 times higher than other cancer centres; CCCs have four times as many clinical trials and eight times the number of Phase I studies. Though perhaps not surprising, these data show that CCCs are key to networks for translational research, clinical studies and outcomes research. But in the Cancer Mission and the EBCP, the EU is expected to not reinvent the wheel. Effective European and national networks of cancer institutions already exist (Box 3) and are accredited by standards (OECI, German Cancer Aid and EACS). Networks such as EORTC and CCE already operate effectively in their spaces. However, at the ground level, ten member states lack even one CCC, and many do not have cancer networks to drive up equalities for patients.

The aims of both the Mission and the EBCP to set up distinct but homogeneous networks of comprehensive cancer infrastructures in every member state are commendable, but the EC needs to be clear what the objective of different networks is; form will follow function. For example, suppose the purpose is to stimulate high‐quality translational, clinical and outcomes research in CCCs (interacting with the EU Knowledge Centre in Cancer). In that case, the EC should issue funding calls for very clear projects to form (or extend) specific networks. Where the purpose is to address inequalities of research and care on the ground within member states, then the EU should issue funding calls for consultancy functions to enable centres and local networks to be formed and improve. Where the purpose is to provide better cross‐border treatment for hard‐to‐treat cancers, then the EC should extend the programme of ERNs to specific cancers, but not to whole CCCs, which would be too diffuse. In many member states, what is required on the ground is consultancy to help organize CCCs and CCC networks effectively around the integration of research and care, multidisciplinarity, standardizing data, samples and outcomes and knowledge exchange. Only in this way, can we address innovation, implementation and inequalities throughout Europe.

Box 5 **The Porto Comprehensive Cancer Centre paradigm**

**Raquel Seruca**, from the Institute for Investigation and Innovation in Health (i3S)/Porto Comprehensive Cancer Centre, Porto, Portugal, gave an overview of the threats and challenges of the Porto Comprehensive Cancer Centre (P.CCC) in collaboration with Carmen Jerónimo, José Carlos Machado, and Rui Henrique. The P.CCC aims to shape and transform the future of cancer care in Portugal. P.CCC encompasses IPO Porto and i3S and seeks to accelerate research and innovation, completing the path of translational research to improve cancer care ultimately. The project is built around fundamental clinical questions in cancer that will be addressed through basic research using a ‘From Bed to Bench and Back’ (B3) concept. P.CCC interacts with the two university hospitals and two medical schools in Porto and is thus part of a vibrant health science hub in the region. P.CCC promotes an open culture and encourages increased collaboration with other research institutes located within the Northern area and other oncology centres, namely IPO Coimbra and IPO Lisbon. Moreover, it will enable sustained engagement with the community to increase public awareness of advances in cancer research and ensure that oncologists have access to clinical trials via a network of affiliated hospitals and primary care centres.

P.CCC’s main ambition is to turn cutting‐edge science into practical benefit for cancer patients, and their families. The latter will be accomplished through exquisite care, innovative research and vital education focused on improving and extending the lives of cancer patients. Novel and more accurate strategies to ameliorate cancer screening, namely in hereditary cancer forms, timely diagnosis, disease monitoring, and understanding cancer dynamics, will be pursued. Furthermore, providing new research opportunities and training to researchers, medical doctors and health professionals will generate a transdisciplinary community with the potential to foster cancer management.

At this stage of development, P.CCC aims to fill Gap 1 of research in Oncology. To implement its objectives and achieve its goals, P.CCC needs to set up several conditions, including (a) sustained funding for cancer research at the national and international level, (b) legitimation of research time for medical doctors, and (c) implementation of research careers and clinical researcher contracts. These actions will contribute to high standards in basic and preclinical research and will translate into groundbreaking clinical research in oncology.

### 4.3 Molecular and Digital Pathology are essential to develop personalized/precision cancer medicine

The crosstalk between molecular and clinical research is bidirectional, and molecular data are currently often required during decision making in the clinical practice. Indications for molecular analysis in pathology have changed over the last decade: while initially performed almost exclusively for diagnostic purposes, molecular pathology is nowadays frequently requested by clinicians for predictive purposes in a significant proportion of patients potentially eligible for targeted therapy. **Fátima Carneiro**, Faculty of Medicine, University of Porto, Porto, Portugal, discussed molecular pathology as an essential complement to conventional morphological tools used to not only obtain a correct diagnosis but also integrate this diagnosis with appropriate assessment of prognosis and prediction of response to therapy [17]. Over the years, WHO classifications of tumours have been transformed from being based on exclusively morphologic criteria to integrating molecular data. This feature is highlighted in the most recent editions of the WHO ‘blue books’ on tumour classification [18].

A close interaction between oncologists and pathologists is necessary for efficient decision‐making strategies, and Molecular Tumour Boards (MTBs; see also next section) represent ideal platforms for a comprehensive discussion of all aspects of molecular diagnostics and the consequences for targeted therapies. A new paradigm for cancer care is emerging that is tailored to the specific genetic profile of an individual’s tumour, regardless of the organ of origin and histological type. In this setting, ‘tumour‐agnostic’ therapies – such as immunotherapies based on the identification of microsatellite instability or high tumour mutational burden [19], or TRK inhibitors, in patients with NTRK fusions that occur in a broad range of different tumour types (e.g., head and neck, salivary gland, bladder, and lung cancers) [20, 21] – can be considered. Even in this scenario, however, interpretation of molecular findings should be made in the setting of pathological features of the tumours (histological type, staging).

Digital pathology, one of the fields of computational pathology [22], includes the process of digitizing histopathology slides and the analysis of the digitized whole‐slide images (WSI) and associated metadata using computational approaches that require adequate infrastructures. In digital pathology, artificial intelligence (AI) approaches have been applied to a variety of image processing and classification tasks, including high‐level tasks such as predicting disease diagnosis, prognosis and treatment response based on morphological patterns, genomic data and immune microenvironment (multiplex immunohistochemistry is getting growing relevance in this field) [23, 24, 25]. Altogether, these approaches allow a temporal/spatial visualization of the evolution/progression of a malignant tumour via histological/genomic/immunological/bioinformatics data integration [26, 27, 28]. Digital pathology is critical to the future of precision medicine when treatment can be tailored to individual tumours in individual patients [24]. The collection of treatment and biological data combined with biobanking provides infrastructures for bidirectional translational research and computational science.

### 4.4 Immunotherapy: an expanding treatment modality

**Laurence Zitvogel**, Gustave Roussy Cancer Campus, Grand Paris, France, reviewed recent progress in cancer immunotherapy (Box 6) and discussed future directions for the field in the context of a Cancer Mission.

L. Zitvogel highlighted the need for launching cross‐border and cross‐cancer prospective surveys with deep cohorts of cancer patients amenable to immuno‐oncology. Such studies, when coupled to comprehensive biobanking during real‐life treatment, will enable large‐scale monitoring of numerous parameters and would require careful data management plan and centralization of data. The coordinated integration and analyses of these metadata will require artificial AI, aspiring to innovate the clinical practice of the therapy of advanced disease (Box 5).

In parallel, accelerated progress cannot be achieved without competitive basic and translational research. The screening of immunogenic cell death‐mediating compounds and monitoring of the patient's tumour cells or metabolites are indispensable for optimizing the synergism of immune checkpoint inhibitors with other compounds, or their bioactivity. In addition, high‐dimensional analysis of the function and regulation of the tumour immune component through the use of spectral flow cytometry, single‐cell RNA sequencing, tissue distribution imaging (digital computerized pathology) and immunodynamics will inform decisions about therapy adjustment (Box 6).

Box 6 **An overview of recent advances in cancer immunotherapy**

The immune system is the hard‐wired host defence mechanism against pathogens and cancer. As such, cancer immunotherapy approaches aspire to harness the immune system for actively combating tumours. Over the past decade, cancer immunotherapy has seen several milestones, including the following:

- The 2018 Nobel Prize in Physiology and Medicine awarded to T Honjo and J Allison ‘for their discovery of cancer therapy by inhibition of negative immune regulation’;
- Sweeping approval of 6 agents by the FDA and EMA to block the PD‐1–PD‐L1 immune checkpoint pathway for the treatment of 16 cancer types;
- A paradigm shift of PD‐1 and CTLA4 blocker use for the management of a broad class of cancers with DNA mismatch repair defect, the first‐ever tissue agnostic approval of cancer drugs;
- Real‐world practice of ‘synthetic immunology’ using adoptive T‐cell therapy with two CD19‐directed chimeric antigen receptor T‐cell products (CAR‐T) for relapsed and/or refractory B‐cell malignancies;
- The emerging new concept of normalizing the mucosal microbiome to modulate systemic immunity.

The impact of the PD‐1/PD‐L1 inhibition on key health outcomes in real‐world situations is remarkable, with estimates reaching a gain of 22 001 life‐years (+31%), 19,073 quality‐adjusted life‐years (+38%) and 22 893 progression‐free survival years (+82%) avoiding 3610 adverse events (−11%) compared with standard of care alone, to be expected over the next 5 years.

Nevertheless, only a 30% of cancer patients receiving immunotherapeutics can benefit from them. Therefore, major efforts have been devoted to unravel the mechanisms underlying sensitivity or tolerance to immunostimulatory or regulatory compounds, develop combined treatments with cytotoxic agents, predict primary resistance, decipher pharmacokinetics, dynamics and toxicities, and to understand on the crosstalk between immunotherapy and metabolism, the gut microbiome or the neural system.

Importantly, to optimize their therapeutic index and economical cost, local injection represents a witty approach still in evaluation. Interception, meaning preventive or prophylactic administration of immune checkpoint inhibitors, has proved to be of great efficacy in preventing distant metastases in stage III melanoma. Finally, the COVID‐19 health crisis combined with the clinical management specifics of immune checkpoint inhibitors has fundamentally shifted the balance between home and hospital care.

Finally, cancer‐associated intestinal dysbiosis, which is possibly linked to chronic inflammatory processes influenced by comedications and cancer therapies, appears to contribute to immunoresistance. Novel diagnostic tools to analyse the taxonomic composition of the gut microbiota are being developed along with microbiota‐centred interventions, and faecal microbial transplantation emerges as an efficient approach to circumvent primary resistance to PD‐1 blockade in melanoma.

In conclusion, the coordination of translational research with the development of top‐level technologies will allow therapeutic breakthroughs in the next‐generation immuno‐oncology (including interception, microbiome studies, and deep cohort research) if the EU complements the efforts to support deep cohorts across frontiers and help mitigate regulatory hindrances.

### 4.5 Liquid biopsies: expanding diagnostic procedures for both therapeutics and prevention

**Olli Kalloniemi**, Science for Life Laboratory, Stockholm, Sweden, described the use of liquid biopsies for detection of cancer and monitoring treatment. Liquid biopsy refers to the detection of DNA, RNA, proteins, vesicles or any other material of potential tumour origin that is secreted or leaked from the tumours to the extracellular space, and hence is detectable in the plasma or other body fluids [29]. Liquid biopsy has the potential to provide a powerful and cost‐effective noninvasive detection method for cancer (Box 7).

Box 7 **The development of liquid biopsies.**

The two most common approaches include detecting circulating tumour cells (CTCs) and circulating free tumour DNA (cfDNA or ctDNA). Both technologies have been studied in over 6000 peer‐reviewed publications over the past decades (PubMed, April 2021) and have reached regulatory approval. Currently, the most dynamically developing area is the cfDNA detection [29], where clinical applications include the following: (a) EGFR driver mutation testing from plasma; (b) diagnosis of driver mutations from plasma when primary tumour sample is not available; and (c) prediction and follow‐up of treatment response during systemic treatments. cfDNA may also help assess the clonal evolution of cancer during therapy, which will be important for future real‐time optimization of therapies. The rise in cfDNA levels may precede radiographic progression by many weeks or months.

The sensitivity of cfDNA technology in cancer diagnosis has been in the range of 50–99%, depending strongly on tumour type and the associated tumour mass and stage of the disease [30]. Importantly, most studies report a 98–99% specificity, which is very high. Changes in ctDNA may therefore already outperform standard tumour marker tests. While cfDNA assays tend to be more specific than traditional protein‐based tumour biomarkers, normal germline DNA must be included in the analysis to exclude clonal haemopoiesis‐derived artefacts and to make sure that one is detecting true cancer‐derived signals [31].

Until recently, the adoption of the cfDNA technology was based on detecting specific changes in the cancer genome by panel sequencing and/or mutation‐specific assays. Whole‐genome sequencing of cfDNA from plasma, with computational analysis to distinguish cancer‐specific signals, has emerged as a powerful alternative [31, 32]. Another recent technology in innovation concerns detecting DNA methylation from cfDNA, using up to millions of CpG sites [33]. This technology offers high sensitivity and the opportunity to predict the tissue of origin, which would be useful for diagnosing cancer of unknown primary.

Even though much more research is needed, evidence already points to the utility of liquid biopsy in translational research, diagnostics and patient follow‐up. Cancer centres, clinical trial groups and funding bodies consider this research seriously and prepare for its large‐scale adoption. Particularly in the early detection and screening of cancer, this technology is also seeing unprecedented commercial interest. If the public sector does not invest sufficiently in research, there is a danger that one or more strong private sector players will dominate the scene, and this could lead to unexpected consequences via, for example, direct‐to‐patient marketing or even direct marketing of screening tests for early detection of cancer to healthy individuals.

### 4.6 Implementation of computational sciences (artificial intelligence)

**Jan Korbel**, European Molecular Biology Laboratory, Heidelberg, Germany, addressed biocomputational approaches to manage the immense amounts of data generated during cancer translational research, clinical research and diagnostics. As a result, computational science in biology, including bioinformatics, modelling, systems and computational biology – often collectively referred to here as Data Science – have become decisive methods relevant to various subfields of cancer research. Indeed, data science will be a key driver for future progress in cancer research, with novel analytical approaches dealing with data of increasing scale and complexity. Data science approaches are likely to foster many clinical research innovations of the future, for example, by jointly integrating information from cancer genomes and automatically analysed pathology images [34, 35]. Data science will also influence and can revolutionize diagnostics by enabling the use of consistent automated or semi‐automated methods to classify clinical/pathological images and will be an essential asset for start‐ups and corporate innovations developing and/or using AI‐based medical products.

Data science approaches in the life sciences depend on coherent data structures, storage and management so that large data sets can be utilized and integrated to exploit their research potential fully. Consequently, the ability to manage, analyse and make growing amounts of biodata widely accessible is of outstanding strategic importance for Europe. The EMBL, with its various sites throughout Europe, is an international infrastructure that has developed a dedicated Data Science programme with a novel approach to life science data management. This programme considers not only the entire data life cycle: from its generation to its analysis, interpretation and publication, but also its archiving, which enables data reuse by the scientific community and the development of novel hypotheses leading to the design of new experiments. The EMBL, in this regard, combines capabilities and service facilities for generating large volumes of high‐fidelity life science data, leading research activities in molecular biology and bioinformatics, and the hosting of widely used data repositories – in a single institution and as an infrastructure for Europe.

The EMBL makes heavy use of its infrastructure for cancer research, such as extracting novel knowledge from internationally shared and high standardized pan‐cancer genomic data sets [36], and exploiting leading‐edge AI technology in cancer research [34]. These developments in data science are for EMBL’s European member states to benefit. For example, the PCAWG data set of standardized cancer genomes has served since its publication last year as an anchoring key reference point for cancer researchers in Europe. The EMBL supports and promotes the open sharing of computational code and publications to foster the reuse of data science and AI technologies. In addition, EMBL encourages the exchange of data for research purposes across Europe, on the premise that data sharing will be key to the realization and success of the future of international cancer research. A key example of this is the European Genome‐Phenome Archive (EGA), which hosts most of the cancer genomics data in Europe, and consented to research data sharing relying heavily on EMBL’s IT infrastructure. Many European countries now have emerging personalized medicine programmes that generate data from national or regional initiatives. Currently, the EGA, a collaboration between the EMBL and the CRG in Barcelona [37], accepts data submissions for data sets consented for research that can be shared across jurisdictions in Europe. Interacting with other European infrastructures, the new Federated EGA will become a distributed network of connected data hubs for sharing human genome data and associated metadata types, including phenotypic data, while complying with national and European data protection requirements. Typically, a hub would be an organization or project that hosts human genetic data so that the data can remain within one jurisdiction, such as the German Human Genome‐Phenome Archive [38] that will act as Germany’s hub within the Federated EGA. By providing a solution to the emerging challenges associated with the secure and efficient management of human genomes and related data, the Federated EGA will promote data reuse and reproducibility and accelerate biomedical research – to foster data sharing as a foundation of applying data science and AI approaches to cancer research across Europe. With regard to the latter, the EMBL has been an early enabler and active supporter of the European Open Science Cloud, which will act as a trusted digital platform for the scientific community, offering access to data and interoperable services to promote data science‐driven solutions in cancer research at an international level.

### 4.7 EC Knowledge Centre on Cancer

**Guy van den Eede** from the European Commission, Joint Research Centre (JRC), Geel, Belgium, emphasized that the cancer challenge is both multifaceted and multidisciplinary, and thus, it requires a holistic approach. The scene set in spring 2020 through the launch of the EBCP (DG SANTE) and the Horizon Europe (2021–2027) Mission on Cancer (DG R&I) requires the alignment, planning and coordination of actions at the scientific and technical level. This task will be undertaken by a neutral, independent yet competent, entity – the EC Joint Research Centre (JRC).

In this context, the JRC is establishing the EC Knowledge Centre on Cancer (KCC) [4]. The KCC will foster a scientific and technical bridge between the EBCP and the Horizon Europe Mission on Cancer, fitting within the new EC Work programme that is entitled ‘Promoting our European Way of Life – Combatting Disease’. KCC will offer already‐established IT systems, gateways, portals, platforms and databases; concrete IT infrastructural components are already in place to unify EC initiatives and actions. The KCC will also offer in‐house competence on cancer prevention, registry data and guidelines and quality assurance for cancer screening, diagnosis and care. The Joint Research Centre, when operating the KCC, will uphold its independence of all private, commercial and national interest. This positions the JRC to play the role of an independent knowledge broker with unquestionable patient/citizen centricity.

The KCC was launched on 30 June 2021, and this occasion coincided with the delivery of the new European Guidelines and Quality Assurance Scheme for Breast Cancer Screening, Diagnosis and Care.

### 4.8 Financing R&D investments in translational research

The issue of financially supporting translational and clinical research through both public and private funding was raised by a panel participant from the pharmaceutical industry.

**Alexander Roediger**, Global Oncology Policy Lead, MSD, Chair EFPIA Oncology Platform, commenting on behalf of EFPIA, stressed that research and development of new treatments follow societal need. The increasing cancer burden is a good example to illustrate this, and R&D has progressed dramatically with new treatment options for cancer patients: between 2012 and 2018, ten new cancer treatments per year were approved by EMA, compared to four per year in the decade before [39]. More importantly, progress for patients has been made: advances in cancer treatments have helped to improve 5‐year metastatic skin cancer survival from 5 per cent to over 50 per cent over the past decade [40].

R&D investment in basic research is often financed through public organizations and private donations that fund research activities at universities. R&D investment at a later stage is commonly financed through private companies that also carry out research activities (clinical trials) in cooperation with the health care sector. However, in 2005, public and private nonprofit funding was about as high as private for‐profit funding. Until 2015 funding from all sources increased, but private for‐profit funding increased the most and accounted for around three‐quarters of total financing (see fig. 90, ref. 39).

A policy environment that encourages collaboration between the private and public sector for investing in translational research will benefit everyone.

The development of and access to new treatments is a collaborative effort. Patient benefit is the result of a healthy ecosystem between private and public, and the pandemic has been a proofpoint for this. Reimbursement by the public is an indicator of societal need, and at the same time an important signal to the private sector’s R&D. Finally, the recent commitment of the European Commission and the member states with EBCP is unique. Such a plan is an important instrument to trigger future R&D through its flagship initiatives [39].

## 5 Infrastructure for Clinical and Prevention Trials

This session focused on the shift of next‐generation clinical trials towards personalized/precision cancer medicine infrastructures for multinational institutional collaboration including stratification of patients for treatment, genomics, imaging, radiation therapy trials, practice changing clinical trials and Molecular Tumour Boards.

### 5.1 Quality assurance of clinical trials structures: harmonization of technical requirements to support multinational institutional collaborations in next‐generation clinical trials

**Denis Lacombe**, EORTC, Brussels, Belgium, discussed the importance of implementing robust clinical research infrastructures that effectively enable access to patients and their biological samples. These frameworks would, on the one hand, offer patients broad access to innovation and affordable, optimized treatments, and, on the other hand, employ multidimensional but adaptive quality assurance standards so that clinical research can rapidly evolve in pace with precision medicine.

Progress in these directions is currently hampered by numerous challenges when a novel drug or technology reaches the clinic. Important barriers surround are as follows: treatment optimization (combination sequence/dosage); treatment de‐escalation (duration/schedule); patient stratification based on robust prognostic/predictive biomarkers; and benchmarking. Added to this complex mix, many other issues relating to the delivery of optimal healthcare include the effective harnessing and storing of data sets, regulatory approval (see also Box 8) and early market access.

Box 8 **Improving regulatory processes during the implementation phase**

**Fátima Cardoso**, Champalimaud Clinical Center/Champalimaud Foundation, Lisbon, Portugal, stressed that minimizing the time lapse between basic research discoveries and introduction of new clinical practice should be a core priority for translational research. This can be addressed by an effective implementation phase, which is currently the bottleneck in making new treatments available to cancer patients.

The current regulatory processes for drug approval in the EU should be tailored, in order to improve outcomes for cancer patients Europe‐wide:

- Improved clinical trial designs and models will be required to bring new drug formulations of decreased toxicity and retained or improved efficacy to the market more rapidly. Noninferiority trials for such formations could be replaced by approaches analogous to those used for approval of biosimilars.
- Regulatory processes allowing for better integration of real‐world evidence and AI analyses with clinical trial data should be prioritized.
- Biomarker‐driven clinical trials should allow simplified data and sample sharing processes across the EU. The MINDACT study, which was one of the very first trials of this kind to be conducted in Europe and funded by the European Commission, highlighted increasing difficulties at this front.
- Cross‐border patient participation in clinical trials should be increased, mainly through the regulation of free patient movement across the EU and of local costs associated with the standard of care.
- Collaboration should be fostered between public and private CCCs, as the latter (given the Champalimaud Clinical Center example) share the qualities and aims of the former.

Calling for a re‐engineering of new models of partnerships in commercial/noncommercial clinical research and clinical trial design – empowered through ‘smarter’ regulatory science and health technology assessment – stakeholders should build on existing solutions and infrastructures (see Box 9 for the SPECTA example) to optimize personalized cancer treatment and care, connect competences and avoid costly duplication.

Box 9 **The SPECTA infrastructure is an example of multicentric collaboration aiming at personalized cancer treatment in Europe**

EORTC has developed a specific infrastructure that embraces medical, ethical and regulatory challenges: SPECTA*, Screening Patients for Efficient Clinical Trial Access*. SPECTA is a collaborative European platform that ensures high‐quality, molecular and pathological screening across several tumour types to aid patient selection for inclusion in clinical trials.

Adopting a patient‐centric approach, this model incorporates quality assurance by design with multidimensional data sets, a multidisciplinary tumour board, integral QC biobanking/access and adaptive methodology to also enable the implementation of new technologies. Regarding regulatory compliance and agility, it has one common protocol for access and project amendments. The preactivation of centres enables speedy access to the SPECTA platform’s projects and resources in order to accelerate the implementation of new clinical trials and advance robust translational research.

An adaptable infrastructure by design, SPECTA, is accessible for patients outside clinical trials and has established a quality‐assured platform for the collection of longitudinal clinicopathologically annotated biological material from cancer patients.

Supporting biospecimen‐based translational research and biomarker discovery with the ultimate goal of offering new therapeutic options to cancer patients, SPECTA is ongoing and has opened in 17 countries across 80 sites, with almost 200 participating institutions, recruiting close to 100 patients each month who are rapidly provided with the genomic make‐up of their disease in order to access optimal, tailored therapies. It also supports the implementation of EU programmes including the Innovative Medicines Initiative (IMI) and the European Reference Network (ERN), as well as developed a new strategy to develop knowledge for rare cancers [46].

Nevertheless, there is still much more to be done in developing and enhancing high‐quality infrastructures for clinical research. Important aspects include complete documentation on the use and proven clinical benefits of novel agents in matched settings, the provision of rigorous data sets that indicate the duration of therapy, optimal dose and combination treatments, and access to rare disease data sets.

The identification of candidate therapies and the assessment of key clinical questions in healthcare practice based on a multinational, independently driven, conditional access system, and the development of therapeutic strategies based on strong scientific rationale, are beyond the remit of commercial pipelines. Very few agents approved by the regulatory agencies truly translate into therapeutic benefits, as has been widely documented in the literature [41, 42, 43, 44, 45]. Backing the innovation that really steps up, namely, the advances that show real benefits in patients and that are readily accessible to all, poses a major challenge. This problem cannot be solved if nonclinically relevant agents continue to exhaust public resources.

D. Lacombe closed by recommending *four key actions*. First, focus should centre on the generation of data sets that document the optimal treatment for cancer patients by integrating clinical research, free of commercial interest in accessing therapies, and inform healthcare systems. Second, new partnership models in commercial/noncommercial research should be re‐engineered based on the continuum of clinical science, regulatory science, and health technology assessment for the optimal treatment and care of cancer patients. Third, public health priorities should command upstream research to implement innovation where needed and avoid multiplication of redundant agents of the same class. Fourth, Europe should build on existing solutions and infrastructures that deliver, optimize competences and avoid costly duplication. Spending time and depleting precious resources without building on existing solutions constitutes a major disservice to patients and society.

### 5.2 Molecular pathology for patient stratification in multicentric clinical trials

**Paolo Nuciforo**, Vall d'Hebron University Hospital and Institute of Oncology, Barcelona, Spain, considered the current complexities and challenges of molecular pathology approaches for therapy selection based on the somatic genotype alterations identified in individual patients.

The number of clinical trials requiring the presence or absence of genomic alterations have soared. In 2017, studies using biomarkers to stratify patients most likely to respond to therapies accounted for around 25% of industry‐sponsored studies. These biomarker‐enriched approaches are increasingly being implemented into clinical trial designs. Currently, patients may be identified for trials and vice versa, with clear pros and cons in both directions.

The adaptive clinical trial design represented by a core study led by VHIO investigators [47] shows how personalized screening strategies for treatment are rapidly evolving in tune with scientific discovery. A dynamic model of biomarker–drug codevelopment in early‐phase clinical trials can help develop clinical studies with agile designs that enrol patients on the basis of multiomics enrichment criteria. This recent study underlines the importance of larger portfolios of therapies that include immunotherapeutic and antibody–drug conjugates with recruitment guided by molecular profiling, as well as progress spurred through major international collaborations and data‐sharing projects such as CCE.

Yet, all is not rosy. There are several major challenges in more effectively implementing and delivering biomarker‐driven precision medicine. First, 65% of consenting patients do not achieve a molecular test result, mainly due to preanalytical issues. In addition, even when results are obtained, not all patients have a targetable alteration. Limited tissue availability is also one of the most limiting factors. The advent of alternative noninvasive approaches including liquid biopsy, however, might well resolve this particular challenge.

Moreover, traditional sampling and processing are optimal for the morphological characterization of tumours, but have not been adapted to the genomic revolution, despite the well‐described, damaging effects of this approach on molecular test results.

Outlining next steps, P. Nuciforo presented the pressing need to go beyond genomic alterations. Digging into the tumour microenvironment using digital spatial profiling (DSP), studying intercellular communication and interaction, and developing an integrative morphology–molecular approach with accreditation of research centres and laboratories that generate molecular test results will be required.

Training opportunities for the next generation of pathologists are also high on the agenda. Investing in education, including novel AI tools for precision oncology, will better prepare pathologists as actors as opposed to mere spectators in this paradigm shift. Considering the current state of the global pathology workforce, fostering networks of translational molecular pathologists should build the necessary critical mass and grow the global workforce in pathology.

Regarding the development of novel, high‐performing treatment decision‐making tools, the Tumour Profiler Study [48] assessed a novel strategy aimed at accelerating diagnostics in parallel with advancing cancer science. This observational study applied and integrated various molecular profiling technologies to create novel opportunities for personalized medicine. It combined a prospective diagnostic approach to evaluate the relevance of in‐depth tumour profiling for support clinical decision making, with exploratory interrogation to advance biological insights into cancer, a potential win–win approach.

### 5.3 Genomics: from discovery to bench‐side genomics

**Emile Voest**, The Netherlands Cancer Institute – NKI, Amsterdam, The Netherlands and Cancer Core Europe, highlighted the need to prioritize comprehensive genomic profiling in order to empower precision oncology.

The tsunami of genomic biomarkers that have been identified and used for drug approvals by regulatory authorities is just the start. We can expect that a multitude of novel therapies (many already in the regulatory pathway) and biomarkers will soon follow. While this is a hopeful message for cancer patients, it also represents a huge responsibility for scientists and clinicians to swiftly translate these new opportunities into meaningful clinical benefits.

Presently, there is a significant time lag, often years, between the approval of novel medicines and the identification of patients who would be most likely benefit from them based on the results generated through the molecular profiling of tumours. As an example, in the Netherlands, even after two years following the approval of the ALK kinase inhibitor crizotinib in 2011 very few patients actually accessed *ALK* fusion testing.

There are several reasons for the delay in the clinical implementation of genomic‐based profiling. Quality controls and validation are time‐consuming, while in cases of rare targets such as *NRG1*, *NTRK*, there is little incentive for testing due to the low ‘hit rate’. These challenges are preventing personalized diagnoses *based on molecular measurements*. In the era of precision medicine, these missed opportunities for patients are unacceptable.

Prioritization of large‐scale genomic profiling at an early disease stage for all patients would help address the above challenges. Signposting towards this direction, unpublished results of an NKI study involving 500 paired biopsies (from 250 patients with metastatic cancer) before and after treatment indicate that therapeutical targets are well preserved over time. Similarly, 95% of actionable variants in clinical trials were found to remain intact, with 92% of genetic changes being detectable at an early stage. This suggests that comprehensive profiling only needs to be performed once over the course of metastatic disease in order to identify treatment opportunities.

In addition to the early identification of actionable targets, the use of approved anticancer medicines could be expanded as an innovative strategy. The Drug Rediscovery Protocol (DRUP) [49] enrols patients that provide informed consent for a pretreatment biopsy for whole‐genome sequencing (WGS) analysis prior to trial participation. DRUP, thus, includes patients with metastatic cancer who have exhausted all other treatment options and present with an actionable molecular profile for which no approved anticancer drugs are available. Each individual case is comprehensively reviewed by an integrated MTB that then stratifies patients over three stages. At the first, unique off‐label drug/tumour type/tumour profile combinations form separate cohorts of 8 patients each, where early signals of activity are investigated. If results show clinical benefit, the cohort expands to 24 patients (stage 2). If successful, this cohort may expand to the stage 3 personalized reimbursement module. If these cases show clinical benefit after 16 weeks’ treatment, costs are reimbursed by payers until disease progression.

DRUP enables the defined use of anticancer drugs beyond their approved label in rare subgroups of cancer, identifies early signals of activity, accelerates the clinical translation of new insights generated by research and creates a publicly available repository of knowledge for future clinical decision making. This model has since been adopted by other European countries whose sister initiatives are now conducting similar clinical trials, to include large‐scale molecular profiling, accelerate the clinical translation of novel insights into the use of anticancer drugs, create publicly available databases for future biomarker‐based research, and offer patients with rare cancers unique treatment opportunities through new avenues.

### 5.4 Modern imaging in oncology

**Regina Beets‐Tan**, the Netherlands Cancer Institute – NKI, Amsterdam, The Netherlands, provided an update on new technologies, approaches and opportunities for cancer imaging. Traditionally, imaging in clinical trials uses computerized tomography for the measurement of tumours. More comprehensive multiparametric imaging can now be performed to capture tumour cell morphology and complete data by diffusion, perfusion and metabolic imaging. Diffusion MRI is already implemented in clinical practice (Box 10).

The design of multicentric trials will necessitate the standardization and harmonization of MRI across the EU. The CCE Imaging Task Force develops and validates quantitative imaging biomarkers in accordance with harmonized cross‐centre imaging guidelines. The Task Force investigators worked together to standardize MRI protocols and assess the variability of scans in phantom and volunteer human studies. Following standardization, they observed that deviation reduced to around 2% and below 10%, respectively. These findings represent a significant development towards implementing novel imaging in clinical trials.

Concerning the predictive powers of molecular imaging, immuno‐positron emission tomography (immunoPET) represents a paradigm shift in the field (Box 10). AI, such as machine learning, neural networks and deep learning, is also showing great promise in more precisely predicting treatment outcomes in individual patients (Box 10).

Box 10 **Latest progress in the field of imaging**

*Diffusion* *MRI*

Being illustrative of how modern imaging can advance novel therapies in a minimally invasive manner, diffusion MRI is already implemented to identify bowel cancer patients with a complete response after preoperative radiotherapy. Signalling exposed by diffusion indicates residual disease, and the absence of a signal corresponds to a complete response. Selection by imaging, combined with clinical data and that of endoscopy, can avoid surgery and improve quality of life for these patients.

Published data on overall survival and disease‐specific survival from the International Watch & Wait Database (IWWD) show a 3‐ to 5‐year survival [50] for bowel cancer patients with complete response after preoperative treatment who were stratified for the Watch & Wait approach. This approach is now being increasingly adopted as a safe and effective alternative to total mesorectal resection (TME) in this specific group of patients.

Current methodology to assess treatment response is based on the measurements of tumour sizes (RECIST) before and after chemotherapy and has its limitations. As an example, RECIST measurement on MRI of a patient with liver metastasis from bowel cancer hardly showed any difference in size (8.3 cm versus 8.1 cm, respectively), suggesting that this patient did not respond to therapy. By extracting the diffusion parameters by diffusion‐weighted imaging (DWI)‐derived apparent diffusion coefficient (ADC), of the same lesion prior to and following treatment, an increase in ADC was observed; indicative of response. This suggests that functional parameters such as ADC values covering the complete tumour can better assess response to treatment and do so much earlier than standard morphology‐based approaches.

The provision of unity system‐specific recommendations for measuring ADC [51] aims to increase the consistency of ADC values acquired and enable large cohort studies for biomarker discovery and the more accurate monitoring of treatment response.

*ImmunoPET*

ImmunoPET combines the superior targeting specificity of monoclonal antibody (mAb) with the inherent sensitivity of the PET technique. Invasive biopsies of lesions performed on a repetitive basis do not accurately capture what is actually happening in the human body, but only the tumour response in isolated tissue. In contrast, immunoPET can differentiate the signal between those patients who are likely to respond to therapy and those who will not. While biopsies provide data on what is happening in one part of the biopsied lesion, immunoPET can capture the entire body and each tumour in its entirety.

*Radiomics*

Recent data [52] show how a noninvasive radiomics signature outperformed standard‐of‐care imaging evaluation in predicting response to immunotherapy at first follow‐up in advanced melanoma and non‐small‐cell lung cancer (NSCLC).

To evaluate whether this novel approach can be used in the multicentric trial setting, CCE’s Imaging Task Force exchanged protocols, fixed the reconstruction parameters and then performed analysis of the liver by radiomics. The investigators found robust, reproducible radiomics signatures across all CCE centres. This supports the potential integration of radiomics‐based evaluation in clinical trials. Future approaches should, however, also consider all biomarkers from tissue and liquid biopsies, as well as genomics, to strengthen prediction models empowered by AI.

Exemplifying the promise of deep learning in clinical trial data analyses, a recently published study [53] assessed the predictive value of a prognostic AI‐Monitor (PAM) for patients with metastatic urothelial cancer receiving immunotherapy. The study investigators hypothesized that quantitative whole‐body prognostic data can be extracted by leveraging AI as a superior and complementary approach to current response evaluation methods.

PAM was designed to identify morphological changes in chest and abdominal CT scans acquired during follow‐up, and correlate these alterations with survival data. Findings showed significant performance in predicting 1‐year overall survival from the date of image acquisition for chest imaging. Subanalysis revealed higher accuracy of abdominal imaging during the first 6 months of treatment, with similar accuracy by chest imaging, 5–11 months after initiating treatment. Compared with current monitoring methods, PAM showed a higher or similar prognostic performance, suggesting its complementary and added value in the clinical setting. These results support the potential, added value of integrating comprehensive AI‐based methodology for prognostic data.

EU’s continued investment in imaging technologies and biomarker‐driven approaches will help to unlock the promise of real‐world evidence, improve outcomes for patients and potentiate anticancer medicines.

### 5.5 Repeated biopsies, biobanking and clinical trials registries with clinical and biological information

**Benjamin Besse**, Gustave Roussy Cancer Campus, Grand Paris, France, discussed the clinical utility of clinical trials employing high‐throughput genomic and transcriptomic profiling of repeated biopsies to more precisely adjust treatment of advanced cancer, as well as biobanking and the development of cancer models from biopsies.

SAFRI02 [54] is an open‐label multicentric randomized phase II trial assessing the efficacy of targeted therapy through the genomic profiling of tissue or liquid biopsies from approximately 1000 patients with metastatic NSCLC.

Another example is the HUDSON [55] ongoing open‐label, multidrug, biomarker‐directed phase II umbrella study for patients with non‐small‐cell lung cancer, whose disease has progressed on immunotherapy. 600 patients across 36 centres (in six countries) were rebiopsied at the time of disease relapse on immunotherapy, and central molecular screening was performed.

Half of the participating patients were assigned either biomarker‐matched targeted therapy plus immunotherapy, or targeted therapy plus immunotherapy, irrespective of biomarker status. This study illustrated that repeated biopsy and biomarker‐stratified trial designs are possible at large scale.

Supported by various pharmaceutical partners, MATCH‐R is an ongoing 9‐year multicohort trial at Gustave Roussy, also designed to identify mechanisms of acquired resistance in patients with advanced cancers who receive treatment with molecular targeted agents and immunotherapy [56]. Preliminary data from MATCH‐R support the feasibility of systematic molecular profiling of tumours acquiring resistance to specific anticancer therapies, ultimately suggesting that one in five patients can receive a newly matched treatment following repeated biopsy profiling.

Moreover, fresh tissue from repeated MATCH‐R biopsies could be used to develop PDX models in one third of a total of 134 patients participating in the study. These unique models will help to develop the next generation of anticancer therapies and advance insights into resistance to therapy.

The US‐based ROS1ders cancer model project [57] was discussed as a second paradigm of how the major challenge of having access to tumour data and biological samples for rare cancers can be addressed. ROS1+ cancers account for around 1–3% of a dozen cancers, including non‐small‐cell lung cancer (NSCLC). ROS1ders, a group of patients and family members dealing with ROS1+ cancer, initiated the above project to provide fresh tumour tissue for creating freely shared cell lines. In addition to doubling the available cell lines for research on ROS1+ cancers, this patient‐driven project highlights the importance of engaging patients to accelerate cancer research (Box 11).

Box 11 **The patients' perspective on clinical trials**

**Bettina Ryll**, Melanoma Patient Network Europe, Sweden, commenting from a patient perspective, indicated that clinical trials exist to serve patients, as stated in the World Medical Association’s (WMA) Declaration of Helsinki. A topic that was not discussed during the Session, the COVID‐19 pandemic, demonstrates the stunning capacity of medical science to identify and combat a major new threat to public health globally. These efforts reflect what can actually be achieved at pace through large‐scale collaboration. While funded medical research in oncology is rapidly advancing the field, and leading to marked improvements in many areas, the actual impact on patients and societies is still not enough, she argued.

Echoing the essence of many of the take‐home messages presented throughout the Session, B. Ryll pointed out that one major challenge is the real‐world broadscale implementation of more potent, personalized treatment strategies and technology‐driven approaches having evidence for application.

Some of the many other issues standing in the way of improving outcomes for cancer patients across Europe include the availability of actual biobanks as opposed to bio archives, prioritizing the biomarkers that can actually make it into validated test in the clinic, and delivering on the General Data Protection Regulation (GDPR) subject‐matter principles and objectives. Discussing the importance of engaging and involving patients throughout the entire cancer research continuum, their integral role in advancing insights into this disease by focusing investigators on the problem that really matters – driving discovery into the clinic for the benefit of all patients, everywhere.

While all above studies indicate the potential of clinical trials involving large‐scale molecular profiling of repeated biopsies, centrally available MTBs are a prerequisite for increasing patients’ access to molecular profiling and matched opportunities for inclusion in downstream academic or industry‐sponsored clinical trials (see also below section on MTBs). Liquid biopsy and fine needle aspiration are noninvasive diagnostic approaches that are both stepping up as valuable clinical tools for molecular profiling in precision oncology [58, 59]. Facilitating repeated biopsy clinical trials, they are showing their worth in identifying therapeutic targets, monitoring response to treatment and establishing molecular mechanisms of drug resistance or sensitivity. Complementing the current suite of technologies and techniques that are shaping the future of personalized cancer care, including omics technologies and immunostaining, these more dynamic and less invasive procedures promise to accelerate the delivery of precision medicine and spur bidirectional translational research.

Considering the strengths of EORTC’s SPECTA platform, academic centres should follow the model of organizing central testing within a biological clinical trial. This approach will greatly facilitate the collection of human biological material and clinical data, as well as enable the Web‐based analyses of results by MTBs.

### 5.6 Development of innovative radiotherapy trials

**Vincenzo Valentini**, Policlinico Gemelli, Rome, Italy, discussed the value of integrating novel radiotherapy and radiomics‐based approaches into multidimensional clinical decision support systems. Technological developments in radiation therapy (RT) focus on three fundamental aspects of RT delivery: targeting; fractionation; and/or irradiation volumes. In the current era of precision medicine, the validation of novel RT technologies should generate real‐world data for therapy prediction by AI technologies.

Over recent years, there has been an explosion in new, even disruptive, technologies in radiation oncology. In some instances, the proven benefit is so great that validation through further studies is not required. As an example, breathing control for the treatment of tumours that are located in moving body regions such as the lungs and liver shows a clear improvement in targeting functionality.

When innovative technology in radiotherapy is not so disruptive, retrospective studies are performed to compare tumour coverage and healthy organ sparing with results from archived images obtained from previously treated patients (Box 12). These *in silico* studies merit regulatory frameworks to evidence the power of their relevance. Evaluation of cost‐effectiveness across different EU national healthcare systems is also required.

Box 12 **Examples of studies assessing novel radiotherapy interventions.**

Outcomes of a retrospective study [61] indicated that dose adaptive magnetic resonance imaging‐guided radiation therapy (MRgRT) in inoperable pancreatic cancer may improve overall survival. Adaptive MRgRT promises an innovative approach to administering higher radiation doses without increasing the risk of acute toxicity, and there is an ongoing prospective clinical trial [62] to further validate outcomes.

In preclinical studies, FLASH radiotherapy has been shown superior to conventional RT in sparing healthy tissues while preserving antitumour activity. Data from a first‐in‐human study [63] confirmed FLASH‐RT as feasible and safe with a favourable outcome in tumour control and reduced toxicity. This novel technology will now need to scale the clinical validation pyramid.

The TRIPOD (Transparent Reporting of a multivariable prediction model for Individual Prognosis Or Diagnosis) Initiative [60] developed a set of recommendations for the reporting of studies developing, validating or updating prediction models. The TRIPOD Statement checklist for the transparent reporting of a prediction model study aims to improve transparency, irrespective of the study methods used (AI, omics, radiomics, etc.). The implementation of an international classification scale and regulatory framework would also be of value in determining the contribution of RT technologies for the exploitation of these models.

The effective integration of radiomics‐based approaches into multidimensional clinical decision support systems also will require standardization processes to reduce methodological and biological variability, ensuring reproducibility in clinical care. The evaluation of RT with real‐world data using AI technology initiates a new era in digitalized RT data generation, integrating context‐sensitive clustered data and advanced predictive models for a more effective personalized cancer medicine practice.

### 5.7 From proof‐of concept trials to practice changing clinical trials

**Elena Garralda**, Vall d`Hebron University Hospital and Institute of Oncology, Barcelona, Spain, addressed current challenges and opportunities in novel clinical trial design and called for innovative infrastructures and platforms to support next‐generation studies in oncology. Current bottlenecks in performing next‐generation sequencing (NGS)‐based trials include patient education issues, acquisition of samples, the analytical validation, high costs, availability and scalability of the NGS assay itself, and the standardization of bioinformatics. Clinical challenges include reporting of data, prioritization and clinical relevance of molecular alterations, the scaling of MTBs outside academia and accessibility to matched therapies.

Dedicated initiatives have been developed to address some of these challenges in clinical trials. The European Society for Medical Oncology’s (ESMO) Scale for Clinical Actionability of molecular Targets (ESCAT) [64] provides a framework for the ranking of genomic alterations as targets for cancer precision medicine. Depending on the level of evidence between an alteration and a given therapy, a grading is given. Importantly, this tool uses a common vocabulary for communication between clinicians and for explaining potential treatment benefits to their patients.

The CCE is a powerful network incorporating seven leading cancer research centres to carry out joint translational and clinical research, conduct next‐generation clinical trials, establish standardized academic diagnostic platforms, create large shared databases and perform outcomes research. CCE’s unique structure has 3 main pillars: clinical and translational research, data analysis centre and education. The first two incorporate multidisciplinary working groups of professionals and technologies. Currently strengthening two major infrastructures, CCE is developing a virtual repository for all tissue samples obtained through its various projects, and a virtual data centre for the sharing of data sets.

Regarding the design of innovative and adaptive clinical trials, two examples were illustrated: the first European multimodular, two‐part academic CCE‐endorsed Basket of Baskets (BoB) study, and the recently launched CCE Building Data Rich Clinical Trials (DART) Consortium, which is supported by EU’s Horizon 2020 research and innovation programme (Box 13).

Box 13 **The BoB study and the DART Consortium exemplify innovative and adaptive clinical trial design**

Led by VHIO on behalf of CCE, BoB [65] is organized in two parts. First, iPROFILER for the molecular profiling of patients from the seven CCE centres, using the same 350 gene capture‐based panel. Depending on the results, patients can be included in the therapeutic part of the study, iBASKET, which comprises different modules and treatments for the different alterations.

BoB represents important advances in the design of clinical studies where patients are prescreened using a homogenized system. Over 600 patients have been screened, and the investigators meet on a weekly basis to discuss the results in these patients through CCE’s MTB Portal [66], which is an innovative support system to guide clinical decision making in precision oncology, built by the Karolinska Institutet, Stockholm (Sweden). This portal enables CCE investigators to perform important correlative studies including the Strategic Immuno‐Monitoring of Patient Therapy (SIMPATHY) [67], for the first module assessing atezolizumab in genomically selected populations. By collecting biopsies including the peripheral blood mononuclear cells (PBMCs), circulating free DNA (cfDNA) and stools samples, this project incorporates cutting‐edge technologies and techniques to perform digital pathology, single‐cell RNA sequencing (scRNA‐seq), imaging mass cytometry (IMC), immune signature analysis and stool analysis, to generate biomarkers for immunotherapy.

BoB shows that once one infrastructure is in place, a second is easier to build. Two years on from the BoB clinical trial, there were still many challenges to resolve. To address ongoing issues, CCE’s Building Data Rich Clinical Trials (CCE – DART) was officially launched earlier this year. Incorporating experts from CCE’s seven European comprehensive cancer centres, along with an additional four non‐CCE partners including SMEs, this EU‐funded, multisite project is a promising example of public–private collaboration to deliver new methods for the design and implement novel, more efficient and effective clinical trials in oncology.

CCE‐DART aims at developing digital tools to facilitate the management of clinical studies and personalized treatment decision making, and statistical designs to potentiate trial methodology. The investigators will also identify and validate new molecular and imaging markers (radiomics) of tumour drug response to treatment, empower patients’ participation in innovative research and ensure sustainability of the project by engaging with pharmaceutical companies and other different stakeholders.

Finally, future action points indicated by E. Garralda included identifying multiple small subsets of patients where specific therapies can achieve high efficacy, developing designs to facilitate small patient populations, resolving the multiple challenges outlined throughout the Summit, and building and strengthening the necessary collaborative infrastructures to collectively improve outcomes for cancer patients.

### 5.8 New concepts and opportunities in cancer prevention clinical trials

**Elisabete Weiderpass**, the International Agency for Research on Cancer (IARC), Lyon, France, stressed the importance of cancer prevention studies in tackling the growing cancer burden in Europe. Cancer prevention clinical trials are the final steps in the extensive research process to confirm the efficacy of new medical advances.

IARC collects and disseminates information about cancer epidemiology, cancer research and the causation and prevention of cancer throughout the world. In addition, IARC performs worldwide clinical trials on cancer prevention (Box 14) and has established strong synergies with EBCP. Focused on implementation research for the successful and effective implementation of vaccination and screening programmes, IARC also integrates new research into social inequalities.

Box 14 **Ongoing IARC cancer prevention trials**

IARC is coordinating important studies in the field of cervical cancer prevention. As an example, recent research, funded by the Bill & Melinda Gates Foundation, assessed the efficacy of a single dose of quadrivalent human papillomavirus (HPV) vaccine against persistent HPV infection compared with 2 or 3 doses.

Findings from the 10‐year follow‐up of the single‐dose vaccinated cohorts compared with an age‐matched unvaccinated cohort in India showed that a single dose of the quadrivalent vaccine could provide protection over a long period of time. Due to the cost‐effectiveness of a single dose, findings from this study will be determinant for the successful implementation of HPV vaccination in limited resource settings. IARC is developing a suite of predictive models to estimate the impact of HPV vaccination in different settings, for example in low‐ and middle‐income countries, and in European countries. These models will support public health decision making and planning at the country level.

Within the framework of a US‐NIH‐sponsored study, IARC worked with a private enterprise to develop a portable, battery‐powered thermal ablator. Thermal ablation has been shown to be simpler to implement than traditional cryotherapy interventions. Historically known as ‘cold coagulation’, this method uses a heated metal probe to destroy abnormal cervical tissue that, if left untreated, may lead to invasive cancer.

The thermal ablator was field tested in Zambia. Women eligible for ablative treatment were randomized into 3 arms to be treated with either thermal ablation, cryotherapy or large loop excision of the transformation zone (LLETZ).

Results showed [69] that the cure rates at 6‐month follow‐up were no different between the treatment arms, thus confirming the effectiveness of the thermal ablation. These findings provide valuable evidence for the World Health Organization (WHO) to recommend thermal ablation as a method to treat cervical precancers to its member states [70].

IARC has recently begun to examine urine‐based HPV testing as a future potential, noninvasive alternative to the current cervical screening method. In collaboration with Lancaster University, UK, researchers have evaluated an infrared spectroscopy methodology to detect HPV in urine. The methodology is simple, and no preprocessing of samples is required. The sensitivity of the test to detect HPV is 84%, and the specificity is 100% (unpublished data).

This proof‐of‐concept study indicates that urine‐based HPV testing could be used as a simple and noninvasive approach to screen for cervical cancer. The accuracy of the model is under development. The implementation of this noninvasive test will lead to increased screening uptake and early detection and therefore decreased mortality from HPV‐related cancers.

The European Code against Cancer (ECAC) [68], coordinated by IARC, is already part of EBCP. The ECAC will be updated to include the latest scientific findings and new evidence‐based recommendations. EBCP aims to make at least 80% of Europeans aware of the ECAC by 2025. This is an example of a great IARC‐EU collaboration to implement successful and impactful cancer preventive measures in Europe.

By strengthening this IARC‐EU cooperation, by identifying opportunities for collaboration and by sharing information, expertise and best practices, more people will avoid developing cancer, more cancer patients will be diagnosed earlier, and more people will suffer less and have a better quality of life after treatment.

### 5.9 The Molecular Tumour Board: a critical infrastructure for delivery of precision medicine

**Júlio Oliveiera**, Portuguese Oncology Institute of Porto (IPO Porto) / Porto Comprehensive Cancer Centre (P.CCC), Porto, Portugal, addressed the importance of implementing MTBs, as knowledge bases and decision support tools for the delivery of precision medicine in oncology. Over the last half decade, around 60 new anticancer medicines have received about 100 new approved indications for more than 20 different tumour types. More than half of these therapies require a recommended testing for pharmacogenomics biomarkers prior to their use. In addition, targeted small molecules and biological treatments account for 90% of the late‐phase oncology pipelines.

Data from a recent study [71] assessing selected molecular profiling initiatives and genotype matching to clinical trials suggest that only around 10% of patients without further standard treatment options were matched to new therapies upon broad genomic testing. Further, a recent report [72] released by the International Quality Network for Pathology (IQN Path), the European Cancer Patient Coalition (ECPC) and EFPIA exposed major inequalities in the availability, quality and reimbursement of biomarker tests in the EU27 and the United Kingdom, and identified country‐specific shortcomings.

Steps must therefore be taken to standardize access to the quality testing of biomarkers across the EU and UK, guaranteeing, at the very least, even a minimum of standard biomarker testing everywhere. To tackle challenges posed by molecular diagnostic testing, including access to NGS and the underlying complexity and heterogeneity of this undertaking, possible solutions include guidelines such as the ESMO published recommendations [73] for the use of NGS in patients with metastatic cancers, more screening programmes in academic research centres and the added clinical value of liquid biopsy.

Access to expert MTBs, including the CCE MTB Portal, as well as to other clinical decision support tools, such as OncoKB [74], which is powered by experts at the Memorial Sloan Kettering Cancer Center, New York, USA, is crucial for the interpretation of variants, multiple targets and germline data, relieving time‐consuming and complex analyses, and avoiding ambiguity and error‐prone tasks.

To address ‘matching’ issues including access to therapies and clinical trials, outcomes evaluation and the heterogeneous regulatory environment, referral networks and collaborative research/data collection will be required, as will the engagement and harmonization of EU regulatory agencies and authorities. Additionally, the IPO Porto centralized MTB, which is open to other healthcare institutions, facilitates the discussion and analyses of cases with the most complex molecular results to more precisely match optimal treatment strategies, as well as offer personalized genetic counselling.

MTBs provide critical infrastructures that facilitate rational, genomic‐driven and evidence‐based personalized treatment recommendations. Importantly, they also engage multidisciplinary teams and advance insights into emerging biomarkers in cancer. They must therefore be valued as must‐have decision‐making platforms in realizing precision oncology.

Finally, as a priority point for the EBCP, all stakeholders in oncology will need to come together to strategically address (and fund) the serious disparities and glaring gaps in access to precision medicine strategies across Europe.

In addition, panel discussions approached the issues of funding schemes to maximize innovation at CCCs (Box 15) and regulatory processes at the implementation phase (Box 8), and also presented the perspectives of patient organizations (Box 11) and the pharmaceutical industry (Box 16).

Box 15 **Funding schemes for maximizing innovation at CCCs.**

**José Carlos Machado**, Institute for Investigation and Innovation in Health V (i3)/Porto Comprehensive Cancer Centre (P.CCC), Porto, Portugal, discussed resources needed to maximize innovation at CCCs.

Research funding opportunities should be tailored to these infrastructures to fuel important initiatives. Public funding of clinical research conducted at CCCs is not currently available through existing framework programmes. Scientific and healthcare bodies should come together, and the respective ministries should collaborate to create new, dedicated funding programmes.

PI‐initiated early clinical studies are very much at the heart of CCCs. These trials constitute a big part of these centres’ added value to the cancer research continuum based on their ‘by design’ capacity to successfully exploit opportunities from fundamental research. PI‐initiated trials thus represent another focus area meriting greater attention, support and development. The CCCs should be increasingly externalized and accessible to other EU countries, for instance, by becoming reference centres for biomarker determination and sharing the expertise provided through dedicated MTBs.

Concerted action for establishing suitable CCC funding programmes is needed and will require a consortium to connect all relevant partners including CCCs, the respective ministries of science and of health, pharmaceutical and in vitro diagnostic companies, and patient advocacy groups. If successfully implemented, this action would represent valuable investment in sustainability and the optimization of resources.

An importantly, focused conversation centred on the crucial role of dedicated, multidisciplinary MTBs in accelerating progress and extending the reach of precision medicine in oncology to an increasing number of cancer patients.

Box 16 **The pharmaceutical industry’s perspective on clinical trials**

**Susan Galbraith**, AstraZeneca, Cambridge, UK, commenting from the pharmaceutical industry´s perspective, stressed in the importance of collaboration and suggested that main focus areas should be prioritized to achieve some impact on outcomes for patients with cancer.

First area of focus is according to S. Galbraith early diagnosis. Only around 30% of cancers are currently diagnosed through screening. With the advent of blood‐based circulating tumour DNA, examples of methylation patterns, improved sensitivity and specificity, this percentage could realistically double.

A second focus area relates to clinical trial designs according to the priorities discussed in the Summit. As an example, recent improvements have been witnessed in the design of lung cancer clinical trials; not only in the metastatic setting but also notably in the neoadjuvant setting; even with the combination of targeted therapies and, potentially, immune‐targeted treatments. The neoadjuvant space creates opportunities to discover much more about each patient’s cancer compared with insights generated by typical phase III trials. As discussed during some of the Session talks, tissue samples can be obtained both before and after therapeutic intervention. This procedure should be embedded in clinical trial designs.

Third, diagnostic standardization should be available for all patients to provide much more value from each diagnosis, with industry partners being valuable actors in this process.

Fourth, while CCCs are critically important for rigour and excellence in clinical trials, in order to overcome the disparities in patients’ access to these studies, high‐quality clinical trials must be made accessible to those who are outside driving distance to their local CCCs. All stakeholders should work together to ensure that future clinical trials can function at distance, which would require data collection at home or close to home, and the digitally supported monitoring of patients. The technology is already there. This will need to be matched by the collective will to comprehensively implement these approaches, and to do so together.

The fifth focus area refers to therapeutically targeting cancer in the early disease setting, while increasingly considering adaptation to treatment and evolutionary responses. Concerning cancers´ multiple mutations and adaptive mechanisms, induction and maintenance regimens should be considered more frequently in multiple solid tumours.

Finally, S. Galbraith reinforced D. Hanahan’s comment on the need of cancer models that incorporate elements of the immune response, so that investigators can better predict response to treatment in the clinic (see also Box 2).

## 6 Infrastructure for outcomes research

In the next session of the Summit, infrastructures were discussed for primary prevention, early detection, assessment of clinical effectiveness, health‐related quality‐of‐life research, survivorship research and health economics.

### 6.1 Outcomes research Infrastructure to support the development of primary prevention

**Joachim Schüz**, International Agency for Research on Cancer (IARC/WHO), Lyon, France, highlighted that primary cancer prevention was identified in both EBCP and the objectives of the European Cancer Mission as a key element in efforts to reverse the trend of an increasing cancer burden in Europe.

Currently known causes and mechanisms of cancer development are estimated to approximately represent half of the causes of the European cancer burden, and the vast majority of cancer cases in Europe, beyond 40%, would be preventable if this knowledge on cancer aetiology was implemented as rigorous primary cancer prevention strategies. However, optimal interventions are still missing for some preventive needs, whereas some interventions do not appear to work as ‘one size fits all’ but require adaption to local cultural, socio‐economical or healthcare infrastructure contexts. To date, almost half of all preventable cancers in Europe are caused by tobacco. Other contributors are the unhealthy diet and lack of physical activity, obesity and alcohol consumption. Smaller but significant contributors are certain infectious agents, occupational hazards, radiations (in particular ultraviolet (UV) radiation and radon) and environmental pollutants [75, 76, 77].

Primary prevention research operates at three levels. At a first level, aetiological, epidemiological and experimental research can identify causes of cancers, the exposure circumstances and pathways, and their relevance at the population level. At a second level, intervention studies are needed to determine how cancer risk can be optimally reduced, especially when behavioural changes are required at either the individual or the group level. In some cases, as in occupational cancers, often technical exposure reduction measures and protection guidelines define successful interventions. Third, implementation research is needed to ensure effective and cost‐efficient implementation in the population, while achieving the highest compliance possible. Although population‐level measures are the ones strictly needed to be implemented by health policymakers, endorsement of prevention recommendations targeted at the individual by policymakers also increase effectiveness. The ECAC assists these efforts by precisely defining what individuals can do to reduce their cancer risk is for the EU countries [68].

Outcomes research monitors the success of primary prevention, primarily, but not exclusively, through close surveillance of cancer incidence and mortality time trends and their geographical variation. Population‐attributable fractions help to quantify the contribution of individual risks to the total cancer burden and also vary over time and geographically. This essential monitoring can build upon existing infrastructures, although there is room for improvement. Most, but not all, of the EU is covered by high‐quality population‐based cancer registries collaborating through the European Network of Cancer Registries (ENCR) and European data collected at the JRC of the EU. The Global Cancer Observatory run by IARC/WHO monitors those data in global context including future projections for optimal cancer control planning. IARC/WHO has also developed the current 4th edition of the ECAC, including provision of the scientific evidence base for which prevention recommendations do reduce the risk of cancer, and its dissemination is mostly driven by the Association of European Cancer Leagues (ECL). European key cancer prevention research institutions work together as the Cancer Prevention Europe (CPE) network providing a platform for future joint European cancer prevention research.

### 6.2 Outcomes research infrastructure to support the implementation of cancer screening

**Partha Basu**, International Agency for Research on Cancer (IARC/WHO), Lyon, France, indicated that the most rigorous evidence for the effectiveness of cancer screening (which could involve a new test or a new cancer site or a novel management approach) is obtained from randomized controlled trials. Robust evidence from multiple studies conducted at different settings can also be combined with favourable results from cost‐effectiveness studies, to provide compelling arguments for implementing new screening interventions in routine health care.

However, the adoption of a new intervention in public healthcare system requires many additional considerations. Novel screening interventions may fail to deliver benefits in real‐life settings, as the latter often fail to reproduce conditions of randomized trials, such as the highly controlled conditions, delivery of the intervention by highly trained staff and concerted efforts to maintain quality across the entire care continuum.

Thus, external validity and feasibility of a new intervention need to be further studied through implementation research (also known as outcomes or health systems research), to ensure that the expected health benefits can be sustainably achieved. Implementation research studies the factors that might influence the final outcome of the evidence‐based intervention, when this is delivered through a routine healthcare system in real‐life settings. Implementation research takes into consideration the building blocks of the health system – governance and coordination, finance, health workforce, infrastructure, service delivery and quality 2assurance. It also aims to assess alternative key determinants of success – acceptability of the screening intervention among the target population and the health providers, the feasibility of achieving a high coverage of the target population when rolled out through routine health services. Thus, implementation research focus on ‘how’ intervention programmes will be implemented in and evaluated.

Implementation research is often undervalued and underutilized. Such research must be conducted with equal scientific rigour and follow same ethical principles as clinical research. The European quality assurance guidelines recommend that any new screening programme should be piloted in the local setting and analysed for cost‐effectiveness prior to scale up. However, there are yet no recommendations on implementation research. The development of an adaptable and adoptable implementation research protocol for cancer screening can easily be achieved through cross‐border collaboration of key institutions.

### 6.3 Outcomes research infrastructure to support the assessment of clinical effectiveness of therapeutic innovations

**Nils Wilking**, Karolinska Institutet, Stockholm, Sweden, in collaboration with Thomas Hofmarcher, Lund University and IHE, Lund, Sweden, discussed how outcomes research infrastructures can address variability in cancer outcomes across Europe, where Western and Northern European countries perform better than Central–Eastern European countries (see Box 3). Adequate health spending on cancer is a prerequisite for achieving high survival rates, but only up to a certain level. Dramatic differences are seen in budgets available for cancer care (measured as direct costs), with highest‐ranked budgets being over than five times higher than lowest‐ranked budgets.

Higher spending strictly correlates with better 5‐year survival rates in some cancers, including lung cancer. However, in other cancers, such as breast, colorectal and prostate cancer, such a correlation is seen up to a certain level of spending, with highest spending conferring no additional improvement in cancer outcomes. Great variation is also registered in health spending on cancer between countries that achieve similar survival rates. Notably, there are many opportunities to improve efficiency and outcomes in cancer care, but we presently lack many tools in order to achieve this improvement.

Bridging the gap between clinical research and healthcare implementation research will help improve cancer outcomes. Whereas clinical trials deliver clinical efficacy, the healthcare needs clinical utility based on information about clinical effectiveness and cost‐effectiveness. Within the Cancer Mission, CCC networks can play an important role in collecting the necessary information needed for assessment of clinical utility of therapeutic innovations as a ‘gate keeper’ before dissemination to the health care. The comprehensive character of national and EU‐wide cancer plans (spanning from prevention to palliative care) and evidence‐based measures coming along with a financing plan are also expected to improve cancer outcomes.

Nationwide population‐based cancer registries consistently recorded in a structured Case Record Form (CRF) across the EU will need to include all essential detail on diagnosis and treatments and be linked to electronic patient records. Relevant CRF indicators should be carefully selected based on scientific evidence and on the ability to reliably and consistently assess them across patients, while also avoiding overlap with other indicators. By the registries, it will be important to identify problems where improvements are needed, in order to argue for actions [39].

### 6.4 Infrastructure support for cancer‐specific health‐related quality‐of‐life research

**Karen Steindorf**, DKFZ/NCT Heidelberg, Germany, explained that cancer‐specific health‐related quality of life, abbreviated as QoL here, is a complex concept covering diverse aspects. Consensus is growing among many stakeholders that clinical (and translational) cancer research should focus more on QoL. Thus, K. Steindorf presented QoL as an ‘add‐on’ to cancer research and an independent research area.

QoL is an intuitive endpoint for randomized clinical trials. This perspective has been taken by patient representatives, as well as by ESMO, who started to include QoL aspects into their formal evaluations of anticancer treatments. The regulatory bodies, such as EMA and FDA, also took positions and explicitly criticized current practice of ignoring or oversimplifying QoL in therapeutic research.

To successfully add the QoL perspective to therapeutic cancer research, requirements for effective assessment of QoL data need to be carefully considered. There is high need for higher‐resolution, systematically assessed data, acquired at meaningful time points with the use of new electronic and/or mobile technologies. Furthermore, the issue of data ownership needs specific attention. Joining forces and expertise from various fields will help addressing many of these complex issues, while it is also expected that there will be no simple and one‐fits‐all solutions. Incorporation of basic QoL assessments in randomized clinical trial designs and in CCC clinical databanks and central cancer registries to generate complete big data repositories may allow major progress.

Furthermore, research focusing on QoL *per se* is still in its infancy, even for fatigue, one of the most common and burdensome symptoms restricting the QoL of many cancer patients for years and decades (including their personal, social and financial situation). Knowledge lags behind, partly because of a lack of profound data. Almost all steps that have been taken to approach therapeutic research need to be also included in QoL research. In the previous example, the aetiology of fatigue, time courses, determinants/predictors, classification of phenotypes/gradings, (risk‐adapted) screening programmes and patient‐tailored management procedures are all major unknowns. Needless to say that the situation is not much better for other QoL parameters, for example sleep quality, cognitive functioning, pain and financial situation. This field has a direct link to cancer care.

In conclusion, there is an immense need for QoL research in two major areas that are closely linked but distinct by their intention. Although the integration of QoL in clinical research is more in the focus of many cancer researchers and clinicians, it may well be that QoL research *per se* is even more promising to advance cancer research. Harvesting the benefit of novel therapies also heavily depends on managing side effects as dose reductions or even treatment terminations may hinder effective drugs to unfold their full potential.

### 6.5 Outcomes research infrastructure for survivorship research

**Francoise Meunier**, Former Head of the EORTC, Brussels, Belgium, in collaboration with Grazia Scocca, Legal Expert, European Cancer Patient Coalition, highlighted that the increase in the prevalence of cancer and new challenges faced by long‐term survivors call for innovative research strategy to improve the quality of life of cancer survivors, and supporting innovative policies. To date, research efforts have focussed more on the diagnosis and treatment of cancer, whereas cancer survivorship has often been overlooked. Importantly in the last years, the number of individuals living after a cancer diagnosis (i.e., cancer prevalence) is growing by approximately 3% annually [78, 79]. Cancer survivors currently represent more than 5% of the overall population in several countries [80]. As reported by the EUROCARE‐6 study and the iPAAC Joint Action, more than 20 million people live after a cancer diagnosis in Europe, marking an increase of + 45% in the period from 2010 to 2020 (13.8–20 million) [81].

Whether being cured (disease‐free) or not, cancer survivors may experience the late and long‐term effects of treatment, emotional distress and fear of tumour recurrence. These effects represent challenges for healthcare systems, which have to ensure appropriate follow‐up care and to promote optimal quality of life: moving from ‘how long’ patients live after diagnosis to ‘how well’ survivors can expect to live from diagnosis onwards. The physical and medical impact (including pain, fatigue, memory problems, lymphedema, infertility, sexual impairment, amputations, secondary malignancies, cardiovascular, pulmonary, renal disease and neuro/muscular impairments) along with the psychological related issues (e.g. depression, anxiety, uncertainty, isolation and altered body image) is not the only problems when it comes to quality of life after having beaten cancer. Increasingly, societal concerns come to light as obstacles for full rehabilitation of cancer survivors, emphasizing the risk of stigma and inequalities. The latter may include not only issues such as changes in interpersonal relationships, and concerns regarding access to financial services, such as health or life insurance and mortgage, but also job lock/loss, or return to school, all new issues that need to be taken into consideration by the cancer research community and, equally, by policymakers and the whole society.

A new strategy to improve and guarantee the quality of life of cancer survivors in Europe should be based on prioritizing three targeted Cancer Survivorship Research and Innovation Pillars (Box 17), which include the perspective of the medical cancer survivorship research, the socio‐economic cancer survivorship research and the politico‐legal cancer survivorship research [82, 83].

Box 17 **The three pillars of targeted cancer survivorship research and innovation.**

**Pillar 1: The medical cancer survivorship research**

The main challenges to improve medical cancer survivorship research involve steps to: (a) integrate cancer survivorship research into cancer research activity in Europe, including specific programmes for children and young adult survivors; (b) improve robust research prioritization using the benefit of data intelligence and long‐term data collection; (c) support interdisciplinary research activity in the survivorship domain and appropriate survivorship research tools; (d) educate the next generation of clinical investigators on the need for long‐term follow‐up; and (e) develop appropriate infrastructures for long‐term medical follow‐up within and outside oncology units and perhaps conduct research to create training for innovative healthcare professionals dedicated to long‐term cancer survivors.

**Pillar 2: The socio‐economic cancer survivorship research**

The key actions to tackle for the socio‐economic cancer survivorship research are as follows: (a) increase knowledge of social determinants contributing to inequalities in cancer survivorship within the EU members states; (b) support research to collect accurate data on the economic burden of cancer – including the return‐to‐work plans for cancer survivors and caregivers; (c) support research to collect data on the impact and cost‐effectiveness of interventions for cancer survivors; and (d) develop multidisciplinary research infrastructure for the integration of social issues into cancer survivorship research activities with an adequate budget.

**Pillar 3: The politico‐legal cancer survivorship research**

Politico‐legal cancer survivorship research needs to: (a) conduct research to improve legal measures and policies to tackle discrimination for cancer survivors; (b) encourage research on the legal aspects of reintegration of cancer survivors back into society, including the return to work and access to financial services; (c) support research conducted by Cancer Patient Advocacy Groups and promote research activities on survivorship for patient empowerment; (d) promote pan‐European research programmes on the right to be forgotten for cancer survivors, based on the existing model of legislation in four EU member states (France, Belgium, Luxembourg and the Netherlands) as it is unfair that not all EU cancer survivors do not benefit from the same absence of discrimination under certain conditions.

A synergy between the cancer research community, the EC, the EU Parliament, national policymakers and cancer patients' organizations will ensure that appropriate and dedicated research on long‐term follow‐up and specific unmet needs of cancer survivors are addressed comprehensively at the EU level. Moreover, in the last years, Europe strengthened its commitment to the fight against cancer at the top of the health and research agenda. In this regard, the implementation of EBCP, particularly with the recommendations from the EU Cancer Mission, represents a unique opportunity to reinforce the efforts to support survivorship–outcomes research cross‐sectionally.

Against this background, cancer survivorship–outcomes research should be recognized as a critical component of the overall cancer research programmes in Europe requiring a well‐defined strategy. The creation and support of outcomes research infrastructures for survivorship research will define critical steps towards full rehabilitation of cancer survivors including full reintegration into society without discrimination. Cancer patients should not have to pay twice! This approach will encourage the recognition of cancer survivorship–outcomes research leading to innovative solutions for long‐term cancer survivors facing numerous new societal challenges and should significantly decrease inequalities and discrimination and promote empowerment for cancer survivors.

### 6.6 Building national and pan‐European infrastructure for cancer health economics

**Bengt Jönsson**, Stockholm School of Economics, Stockholm, Sweden, emphasized that health economics should be an integrated part of the cancer research continuum so that outcomes from interventions with different costs and outcomes can be optimized. While treatment choices need to be made across a rapidly growing number of alternative interventions, the building of national and pan‐European infrastructures for health economics must be prioritized.

Health economics is well established as a research topic in cancer, but resources and applications are scattered and there is a need for core‐funded health economics units attached to CCCs in each EU country. This will create an infrastructure for professional development and international collaboration in this research domain. The links to CCCs will allow for a collaborative and timely design of comparative studies investigating relative effectiveness and cost‐effectiveness. Timely performed studies can produce evidence when decisions on payment and use of new interventions are needed, just before new interventions enter clinical practice. Systematic cost‐effectiveness studies are important, but there is also a need for developing models for simulating cost‐effectiveness of alternative actions in prevention, early detection, diagnosis and treatment, based on best available clinical and epidemiological evidence.

In addition, early evaluation for coverage decisions, including prices and payment mechanisms, is important for broadening patient access to new interventions. Waiting until market entry is commonly too late to exert meaningful influence on uptake and use of new methods.

An infrastructure for health economics also needs to include real‐world epidemiological, health systems and services and outcomes data. For rare cancers, including childhood cancers, pan‐European approaches are needed to collect epidemiological data with information about interventions and relevant patient characteristics, including molecular characterization. Patient‐relevant outcomes data such as mortality, survival, QoL, and patient‐reported outcomes, as well as health services and systems data of good quality, are necessary for development and evaluation of cancer policies.

Building an infrastructure for health economics finally requires the possibility to extract economic data from hospitals at a national level, covering all modalities, site‐specific cancers and across the entire patient pathway. This will include data on resource use for defined patients and episodes of care, data on costs for preventive, diagnostic, surgical, and radiological interventions and cancer medicines, and data on indirect costs and costs for formal and informal care for patients at different stages of the disease.

### 6.7 Value‐based frameworks for outcomes implementation and measurement

**Marta Soares**, Portuguese Oncology Institute of Porto, (IPO Porto) / Porto Comprehensive Cancer Centre (P.CCC), Portugal, introduced how value‐based frameworks can support the improvement of health results, using the IPO Porto lung cancer case study (the FAROL Project, Box 18) as a paradigm. Value‐Based Health Care (VBHC) aims at creating value for all health system stakeholders: improving the relationship between the quality of care and the required costs. The value equation is a tool to measure value: patient health outcomes achieved (clinical and patient‐reported) divided by the cost of delivering those outcomes.

Box 18 **A case study: outcomes implementation and measurement in lung cancer**

Lung cancer leads the causes of cancer death and has been related to a significant DALY (Disability‐Adjusted Life Years) loss over the past years. In fact, lung cancer survival rates drop as disease progresses, urging the need for early diagnosis and a robust disease management. Today, healthcare institutions are reshaping their way of work, progressively combining clinical practice improvements with information technology evolution. IPO Porto/ Porto Comprehensive Cancer Centre (P.CCC) has partnered up with several institutions (APAH, ROCHE, IQVIA, ICHOM, All. Can) to develop a value‐based healthcare pilot project in lung cancer – the FAROL Project.

The FAROL Project required the definition of the most accurate care pathway, to enable cost assessment and outcomes measurement. The first step was to define the lung cancer pathway: select the medical condition, define the care delivery value chain and develop process maps that include each activity in patient care delivery. Then, the lung cancer cost assessment per patient was defined. For that, the time‐driven activity‐based costing methodology was used. Time estimates were obtained for each procedure (activity and resource), the cost of supplying patient care resources (direct and indirect resource) and each resource’s capacity was estimated to calculate capacity cost rate, and total cost of patient care was calculated. Outcomes measurement implementation follows a stepwise approach starting with outcomes definition, followed by data collection, analysis and benchmark.

Outcomes measurements may rely on the existence of already‐defined and well‐documented standard sets. ICHOM has defined the standard set for lung cancer, with suggested tools for patient‐reported outcomes, and proposed the evaluation time frame. Since not all outcomes were systematized at IPO Porto, a subset of variables was selected for further analysis: survival rate; time between diagnosis and treatment initiation; percentage of patients under pharmacological treatment in the last 30 days of life; days spent in hospital in the last 30 days of life; percentage of unplanned hospitalizations upon treatment definition; and percentage of unplanned appointments upon treatment definition.

The implementation of VBHC brings tangible benefits to patients, healthcare practitioners and organizations, namely: (a) promotes standardization and reduces variations in patient care, (b) ensures a patient‐centric care delivery, (c) fosters the communication and involvement of clinicians in management decisions, (d) improves resource capacity utilization, (e) optimizes the full cycle of care with the elimination of processes that do not add value to the care delivery chain, (f) provides a means of continuous quality improvement, (g) supports the improvement of the patient experience in the care delivery process and (h) helps to improve clinical outcomes.

VBHC is highly dependent on intrinsic institution‐based factors, which are all present at IPO Porto, including leadership, care organization, healthcare professional’s involvement, incentives and a proper IT system. The FAROL Project has several benefits: from better understanding of patient pathway to financial efficiency and quality of care improvement.

Taken together, despite focusing on diverse aspects, all speakers of this session highlighted the need for establishing robust, multicentric infrastructures for coordinated and structured research in outcomes research, a message that was further corroborated during panel discussions (Box 19).

Box 19 **Panel discussions on infrastructures for outcomes research**

**Jose M Martin‐Moreno**, University of Valencia, Valencia, Spain, reflected on the importance of investing in infrastructures for outcomes research within the field of cancer prevention and control – a highly dynamic field with a process cycle that must be comprehensively addressed.

JM Martin‐Moreno noted that ‘we should be prepared to respond to why we do not prevent what we know how to avoid’. Feasibility and external validity should complement randomized clinical trials for ensuring the success of new screening strategies. In addition, cancer registries should improve the registration of detailed info on diagnosis and treatments through the consistent use of a structured Case Record Form across the EU. Finally, quality of life should be, beyond simple survival, the ultimate goal of cancer treatment in all EU healthcare systems and beyond through the evaluation of perspectives arising from the medical cancer survivorship research, the socio‐economic cancer survivorship research and the politico‐legal cancer survivorship research. The enormous challenge of trying to prevent and control cancer by promoting health is one of the noblest and most exciting missions in our society.

**Matti Aapro**, ECCO, Brussels, Belgium, told the audience that ECO was pleased to contribute to the European Cancer Research Summit and support its associated ‘Porto Declaration’. Via the EBCP and the Cancer Mission, the EU states and citizens are presented with a key opportunity to lead the world in many areas, including translational research, clinical research, and outcomes research. Whether it is about HPV‐related cancer elimination, creating a tobacco‐free Europe or setting hard targets on access to screening or comprehensive cancer centres, it is encouraging to see such objectives stated and committed to. These objectives will enable measurement of success and the planning of strategies for improving shortcomings. Now, the energy must shift from consultation to implementation. Nevertheless, as with any cross‐border endeavour, the infrastructure for delivery needs to be carefully developed. The Porto Declaration, as the major outcome of the European Cancer Research Summit, describes the key lines along which all stakeholders need to jointly work together to ensure results without major delays.

## 7 Cancer screening programmes in the EU

**Eva Kondorosi**, European Commission's Group of Chief Scientific Advisors, Brussels, Belgium, informed the audience about the EU screening programmes, which aims at detecting cancer at an early stage to improve health outcomes. Early detection of cancer can be done through population‐based screening programmes, testing the targeted population at risk or testing of people in a clinical setting. Although new technologies and personalized medicine strategies can improve screening programmes, there is much space for optimization and translation into practice.

The identification of new biomarkers, the development of tests for known genetic mutations that mark cancers with late clinical presentation (oesophagus, pancreatic and ovarian) and the validation of the above through population‐based screening programmes will require vigorous assessment of the current and new diagnostic technologies in the EU.

In December 2003, the EU Council published recommendations [84] for population‐based screening programmes using conventional techniques for cervical, colorectal and breast cancers [85]. These recommendations were instrumental in ensuring that the vast majority of people in selected target age ranges in the EU member states have access to organized screening programmes. As of 2020, 25 EU member states had introduced in their National Cancer Control Plans population‐based screening programmes for breast, cervical and colorectal cancer. However, many programmes have not been fully implemented yet, and inequalities persist within and between member states [8]. For example, coverage of the target population ranges from 6% to 90% for breast cancer screening and from about 25% to 80% for cervical cancer screening [6]. Therefore, the EC will make a proposal by 2022 to update the Council Recommendation on cancer screening according to the most current scientific evidence. If supported by scientific evidence, cancer screening will be extended beyond breast, colorectal and cervical cancer over to prostate, lung, gastric and other cancers. The EC Group of Chief Scientific Advisors will provide advice to assist this work, focusing on three specific scientific questions:

- How can screening programmes targeting breast, cervical and colorectal cancers be improved throughout the EU?
- What is the scientific basis for extending such screening programmes to other cancers, ensuring their feasibility throughout the EU?
- Which are the main scientific elements to consider, and best practices to optimize risk‐based cancer screening and early diagnosis throughout the EU?

The advice, which will be issued to the Commission in February 2022, will also benefit from an evidence‐based review report on cancer screening prepared by SAPEA, a consortium that brings together the outstanding knowledge and expertise of Fellows from over 100 Academies, Young Academies and Learned Societies in over 40 countries across Europe [86]. The Federation of European Academies of Medicine (FEAM) will be the lead academy network in the SAPEA consortium, which will provide the Evidence Review Report based on the available scientific knowledge serving as a knowledge source for the scientific opinion of the Group of Chief Scientific Advisors on Cancer Screening.

## 8 Strategic development of precision cancer medicine in the United States

**Richard Schilsky**, Past President and former Chief Medical Officer of the American Society of Clinical Oncology, Alexandria, VA, USA, presented an overview of the current developments of precision cancer medicine in the United States and highlighted that even though its implementation in the United States is technologically far advanced, there remain many barriers that need addressing. The full presentation was recently published in the July Issue of Molecular Oncology [87].

## 9 Closing remarks

The role of the Horizon Europe missions in establishing significant synergies and linkages between the EU and national policy initiatives, research and care infrastructures, and funding programmes is crucial, as also stressed by **Patrik Child**, Deputy Director‐General of Research & Innovation, European Commission, Brussels, Belgium. P. Child indicated that the missions could also reach out and stimulate the mobilization of other programmes, including the recovery–resilience plan in the post‐COVID‐19 era.

According to P. Child, the Mission on Cancer will be able to expand dedicated infrastructures for translational cancer research, cancer control and real‐world data, ensuring the establishment of connected, high‐quality networks as these were outlined at the Summit. For example, the proposed EU platform UNCAN.eu (understanding of cancer), the networks of comprehensive cancer infrastructures and the European Patient Digital Centre (accelerating progress in research and cancer control using a patient‐centred approach) could be parts of this network.

By integrating with the EBCP, these infrastructures will become part of a broader ambitious EU agenda, which will require the strong collective commitment of numerous actors, including member states and citizens. As a result, P. Child welcomed the Porto Declaration as the first step towards this strong commitment to sharing infrastructures.

**Simona Kustec**, Minister for Education, Science and Sport, Slovenia, highlighted another aspect of the Porto Declaration, presenting it as the outcome of the political determination of the Trio Presidency of Germany, Portugal, and Slovenia to achieve a continuous and coherent cancer policy across Europe for high‐quality research, equity and inclusivity. S. Kustec noted that the Slovenian Presidency will follow the Trio Presidency principles of inclusiveness, political ownership and commitment, coherence and agility, and called all EU member states to endorse the declaration by actively engaging cancer patients, cancer survivors and their families to transform research priorities, as well as by collaborating to ensure accessibility for all European regions to joint international, multicentric clinical studies.

**Manuel Heitor** stressed that the *Porto Declaration on Cancer Research* of May 2021 calls for a collective action throughout Europe towards a comprehensive translational cancer research approach focused on personalized and precision medicine and covering the entire cancer research continuum. Specific actions are required to strengthen a network of well‐distributed and interconnected high‐quality infrastructures for translational research, clinical and prevention trials and outcomes research, to ensure that science‐driven and social innovations benefit patients and individuals at risk across the healthcare systems in the European Union (EU).

The declaration has been framed by the discussion that such a European‐wide deployment of high‐quality infrastructures has the potential to achieve in 2030 a 10‐year cancer‐specific survival for 75% of patients diagnosed in EU member states with a well‐developed healthcare system.

In this context, the European Cancer Research Summit emphasized that broadening the social basis for knowledge‐based activities in cancer treatment and prevention, and strengthening the research system producing new knowledge and excellence, should be combined with fostering intermediaries with society and the economy at large. This will require a focusing on the continuous skill development for researchers, clinicians and teaching staff throughout the entire education, research and healthcare systems. In addition, establishing close links between professionals and the society will be a continuous process based on a clear understanding of science–society relationships, and expanding beyond the currently dominating policies that consider science only through short‐term, demand‐driven economic development issues.

An effective Cancer Mission in Europe will help reduce the gap between science and policy, mainly by actively involving in policymaking the cancer research and cancer healthcare stakeholders and cancer patient communities. Only through such an approach can specific scientific and diversified local issues be aligned into an overall strategy with practical relevance to all European citizens [10].

The Summit discussions presented above suggest that major tasks should be conducted at a European‐wide level in association with the need to promote periodic research assessments of CCCs in Europe. This will significantly impact the building‐up of capacities and institutions throughout Europe and should be implemented in association with a properly defined cancer mission.

As a final remark towards the emerging debate on the future of Europe, the coevolution of human capital formation and research capacity in its various forms (i.e., academic, translational and clinical) is critical to promote the absorptive capacity required, so that regions and countries throughout Europe can learn how to use science to effectively improve the quality of life.

## Conflict of interest

Where authors are identified as personnel of the International Agency for Research on Cancer/World Health Organization, the authors alone are responsible for the views expressed in this article and they do not necessarily represent the decisions, policy or views of the International Agency for Research on Cancer/World Health Organization. JT reports personal financial interest in form of scientific consultancy role for Array Biopharma, AstraZeneca, Avvinity, Bayer, Boehringer Ingelheim, Chugai, DaiichiSankyo, F. Hoffmann‐La Roche Ltd, Genentech Inc, HalioDX SAS, Hutchison MediPharma International, Ikena Oncology, IQVIA, Lilly, Menarini, Merck Serono, Merus, MSD, Mirati, Neophore, Novartis, Orion Biotechnology, Peptomyc, Pfizer, Pierre Fabre, Samsung Bioepis, Sanofi, Seattle Genetics, Servier, Taiho, Tessa Therapeutics and TheraMyc. And also educational collaboration with Imedex, Medscape Education, MJH Life Sciences, PeerView Institute for Medical Education and Physicians Education Resource (PER). JT also declares institutional financial interest in form of financial support for clinical trials or contracted research for Amgen Inc, Array Biopharma Inc, AstraZeneca Pharmaceuticals LP, BeiGene, Boehringer Ingelheim, Bristol Myers Squibb, Celgene, Debiopharm International SA, F. Hoffmann‐La Roche Ltd, Genentech Inc, HalioDX SAS, Hutchison MediPharma International, Janssen‐Cilag SA, MedImmune, Menarini, Merck Health KGAA, Merck Sharp & Dohme, Merus NV, Mirati, Novartis Farmacéutica SA, Pfizer, Pharma Mar, Sanofi Aventis Recherche & Développement, Servier, Taiho Pharma USA Inc, Spanish Association Against Cancer Scientific Foundation and Cancer Research UK. MB has received funding for his research projects and for educational grants to the University of Dresden by Bayer AG (2016‐2018), Merck KGaA (2014‐open) and Medipan GmbH (2014‐2018). He is on the supervisory board of HI‐STEM GmbH (Heidelberg) for the German Cancer Research Center (DKFZ, Heidelberg) and also member of the supervisory body of the Charité University Hospital, Berlin. As former chair of OncoRay (Dresden) and present CEO and Scientific Chair of the German Cancer Research Center (DKFZ, Heidelberg), he has been or is responsible for collaborations with a multitude of companies and institutions, worldwide. In this capacity, he has discussed potential projects and signed contracts for research funding and/or collaborations with industry and academia for his institute(s) and staff, including but not limited to pharmaceutical companies such as Bayer, Boehringer Ingelheim, Bosch, Roche and other companies such as Siemens, IBA, Varian, Elekta, Bruker, etc. In this role, he was/is also responsible for the commercial technology transfer activities of his institute(s), including the creation of start‐ups and licensing. This includes the DKFZ‐PSMA617 related patent portfolio [WO2015055318 (A1), ANTIGEN (PSMA)] and similar IP portfolios. MB confirms that, to the best of his knowledge, none of the above funding sources were involved in the preparation of this paper. BB has received research funding from 4D Pharma, Abbvie, Amgen, Aptitude Health, AstraZeneca, BeiGene, Blueprint Medicines, BMS, Boehringer Ingelheim, Celgene, Cergentis, Cristal Therapeutics, Daiichi‐Sankyo, Eli Lilly, GSK, Inivata, Janssen, Onxeo, OSE immunotherapeutics, Pfizer, Roche‐Genentech, Sanofi, Takeda, Tolero Pharmaceuticals. FC declares consultancy role for: Amgen, Astellas/Medivation, AstraZeneca, Celgene, Daiichi‐Sankyo, Eisai, GE Oncology, Genentech, GlaxoSmithKline, Macrogenics, Medscape, Merck‐Sharp, Merus BV, Mylan, Mundipharma, Novartis, Pfizer, Pierre‐Fabre, prIME Oncology, Roche, Sanofi, Samsung Bioepis, Seagen, Teva. SF is a consulting or advisory board member at Bayer, Illumina, Roche; has received honoraria from Amgen, Eli Lilly, PharmaMar, Roche; has received research funding from AstraZeneca, Pfizer, PharmaMar, Roche; has received sponsorship of travel or accommodation expenses by Amgen, Eli Lilly, Illumina, PharmaMar, Roche. SG owns AstraZeneca stock and is a full‐time employee of AstraZeneca. PN has had an advisory role at Bayer, MSD Oncology, has received honoraria from Bayer, Novartis and MSD Oncology, and has had travel expenses paid by Novartis. JO has been an advisory board member at Roche, Novartis, Bayer, Merck, Eisai, Astrazeneca, Pierre Fabre Medicament and Bristol‐Myers Squibb. He has also received research funding by IPO Porto, Astrazeneca, Fundação para a Ciencia e a Tecnologia (FCT) and Liga Portuguesa Contra o Cancro (LPCC). AR is an employee of European Federation of Pharmaceutical Industries and Associations, Brussels, MSD International Business GmbH, Kriens, Switzerland[CvG1], and Merck Sharp & Dohme Corp., a subsidiary of Merck & Co., Inc., Kenilworth, NJ USA, who may own stock and/or hold stock options in the Company.RS serves as principal investigator of the ASCO TAPUR study. ASCO receives research grants from the following companies in support of the study: Astra‐Zeneca, Bayer, Boehringer‐Ingelheim, Bristol Myers Squibb, Genentech, Lilly, Merck, Pfizer, Seattle Genetics. Dr. Schilsky serves as a member of the managing board of Clariifi and as a consultant to Bryologyx, Cellworks Group, EQRx, and Scandion Oncology. The Netherlands Cancer Institute receives research support via EV from Roche, Astrazeneca, Eisai, Novartis, GSK, Clovis, BMS, MSD, Pfizer, Amgen, Bayer, Lilly, Janssen and Seagen. LZ is founder of everImmune, member of the board of directors of Transgene, member of the scientific advisory board of Transgene, EpiVax, Lytix Biopharma. LZ has also had research contracts with: Merus, Roche, Tusk, Kaleido, GSK, BMS, Incyte, Pileje, Innovate Pharma, and Transgene and has received honoraria by Transgene. All other authors have no conflicts of interest to declare.
